# Bassoon contributes to tau-seed propagation and neurotoxicity

**DOI:** 10.1038/s41593-022-01191-6

**Published:** 2022-11-07

**Authors:** Pablo Martinez, Henika Patel, Yanwen You, Nur Jury, Abigail Perkins, Audrey Lee-Gosselin, Xavier Taylor, Yingjian You, Gonzalo Viana Di Prisco, Xiaoqing Huang, Sayan Dutta, Aruna B. Wijeratne, Javier Redding-Ochoa, Syed Salman Shahid, Juan F. Codocedo, Sehong Min, Gary E. Landreth, Amber L. Mosley, Yu-Chien Wu, David L. McKinzie, Jean-Christophe Rochet, Jie Zhang, Brady K. Atwood, Juan Troncoso, Cristian A. Lasagna-Reeves

**Affiliations:** 1 Stark Neurosciences Research Institute, Indiana University School of Medicine, Indianapolis, IN USA; 2 Department of Anatomy, Cell Biology & Physiology, Indiana University School of Medicine, Indianapolis, IN USA; 3 Department of Pharmacology and Toxicology, Indiana University School of Medicine, Indianapolis, IN USA; 4 Department of Biostatistics and Health Data Science, Indiana University School of Medicine, Indianapolis, IN USA; 5 Department of Medicinal Chemistry and Molecular Pharmacology, Purdue University, West Lafayette, IN USA; 6 Purdue Institute for Integrative Neuroscience, Purdue University, West Lafayette, IN USA; 7 Department of Biochemistry and Molecular Biology, Indiana University School of Medicine, Indianapolis, IN USA; 8 Division of Neuropathology, Department of Pathology, Johns Hopkins University School of Medicine, Baltimore, MD USA; 9 Department of Radiology & Imaging Sciences, Indiana University School of Medicine, Indianapolis, IN USA; 10 Center for Computational Biology and Bioinformatics, Indiana University School of Medicine, Indianapolis, IN USA; 11 Department of Medical and Molecular Genetics, Indiana University School of Medicine, Indianapolis, IN USA

**Keywords:** Neurodegeneration, Alzheimer's disease, Synaptic transmission

## Abstract

Tau aggregation is a defining histopathological feature of Alzheimer’s disease and other tauopathies. However, the cellular mechanisms involved in tau propagation remain unclear. Here, we performed an unbiased quantitative proteomic study to identify proteins that specifically interact with this tau seed. We identified Bassoon (BSN), a presynaptic scaffolding protein, as an interactor of the tau seed isolated from a mouse model of tauopathy, and from Alzheimer’s disease and progressive supranuclear palsy postmortem samples. We show that BSN exacerbates tau seeding and toxicity in both mouse and *Drosophila* models for tauopathy, and that BSN downregulation decreases tau spreading and overall disease pathology, rescuing synaptic and behavioral impairments and reducing brain atrophy. Our findings improve the understanding of how tau seeds can be stabilized by interactors such as BSN. Inhibiting tau-seed interactions is a potential new therapeutic approach for neurodegenerative tauopathies.

## Abstract (web summary)

Martinez et al. identified the protein interactome of the tau seed responsible for propagation. The authors found how the presynaptic protein Bassoon interacts with the tau seed enhancing its stability and subsequently tau toxicity and spreading.

## Main

Pathological aggregation of tau is a defining histopathological feature of Alzheimer’s disease (AD), progressive supranuclear palsy (PSP) and many neurodegenerative diseases known as tauopathies^1,2^. A major focus of research has been to understand the propagation of pathological tau. Despite the knowledge acquired, the cellular mechanisms involved in tau propagation remain unclear, although reports have linked it to synaptic activity^3–5^. Tau released due to neuronal activity could be impaired by blocking presynaptic vesicle release^3^, suggesting that tau is released via presynaptic compartments. However, the nature of the tau species involved in tau spreading and the precise seeding mechanism and template remain unclear. Despite this uncertainty, some studies suggest that a rare species of soluble high-molecular-weight (HMW) tau is involved in trans-synaptic propagation^6,7^. Whether these HMW tau-containing particles exclusively comprise tau or contain other constituents, such as proteins or lipids for propagation, is unknown.

Numerous tau interactome-based studies have established that tau interacts directly with proteins and complexes involved in various biological functions in addition to those associated with microtubule stability^8–14^. Studies have also identified interactors of total tau in tauopathy mouse models^15–18^. Other studies have examined the interactome of total tau in human cell lines^19,20^ and induced pluripotent stem cell-derived neurons^21^. However, no studies have directly compared the interactomes of seeding-competent tau with those of monomeric tau or determined how tau-seed interactors affect the nature of this seed and, subsequently, tau propagation.

Here we performed unbiased quantitative mass spectrometry to characterize the tau species involved in tau propagation and identify proteins that specifically interact with the tau seed. Our results established that only a slight fraction of total tau in the brain forms a HMW complex with strong seeding activity. This tau seed interacts with proteins that do not interact with tau monomers. Bassoon (BSN), a scaffolding protein of the presynaptic active zone^22^, was identified as an interactor of this tau seed. We also identified BSN as an interactor of tau seeds isolated from AD and PSP postmortem samples. Our results demonstrate how BSN exacerbates tau seeding and toxicity in vitro and in vivo. Furthermore, BSN downregulation decreases tau spreading and rescues synaptic and behavioral impairment in a mouse model of tauopathy. Our findings highlight the importance of identifying tau interactors, such as BSN, that could act as stabilizers of the tau seed, enhancing its propagation and toxicity.

## Results

### BSN protein interacts with a high-molecular-weight tau seed

**Fig. 1:**
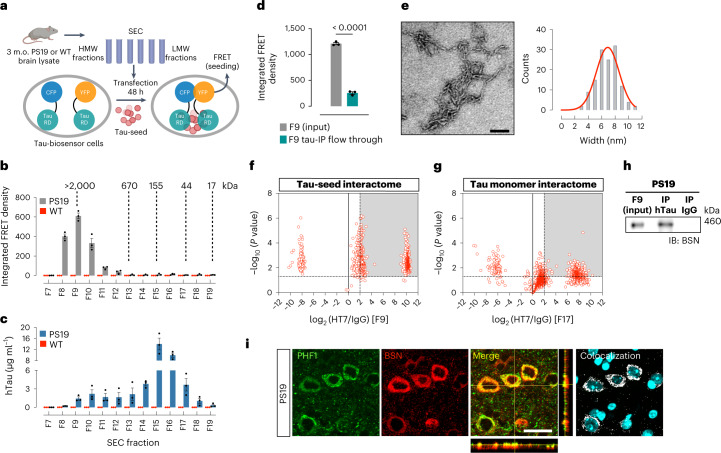
A high-molecular-weight tau seed interacts with BSN protein. **a**, Schematic of the tau-seeding assay of SEC fractions. **b**, Tau-seeding activity of SEC fractions obtained from 3-month-old PS19 and WT mouse brain lysates. **c**, Total human tau (hTau) detected by ELISA in SEC fractions from PS19 and WT mouse brain lysates. **d**, Tau-seeding activity of the PS19 SEC fraction 9 (F9) containing HMW tau, before and after hTau IP using HT7 antibody. **e**, EM of the F9 IP product showing short tau filaments, and width distribution of these filaments. Scale bar, 100 nm. The experiment was repeated three times with similar results. **f**,**g**, Volcano plot indicating tau interactors found in F9 (**f**) and F17 (**g**), identified by liquid chromatography coupled to tandem mass spectrometry (LC–MS/MS). **h**, Western blot of BSN from co-IP of human tau (HT7) from F9 in PS19 brain lysates. **i**, Representative immunofluorescence in PS19 mice cortex for pathological tau (PHF1) and BSN protein. Colocalized pixels are shown in white, whereas nuclei are visualized in cyan. The merged panel includes orthogonal images of reconstructed three-dimensional views. Colocalization analysis was performed to determine pixel intensity correlation between PHF1 and BSN. Scale bar, 25 µm. Data are shown as the mean ± s.e.m. Experiments were performed in triplicates (**b**–**d**, **h** and **i**), and significance was determined by an unpaired two-tailed Student’s *t*-test (**d**). CFP, cyan fluorescent protein; YFP, yellow fluorescent protein. Source data

**Extended Data Fig. 1:**
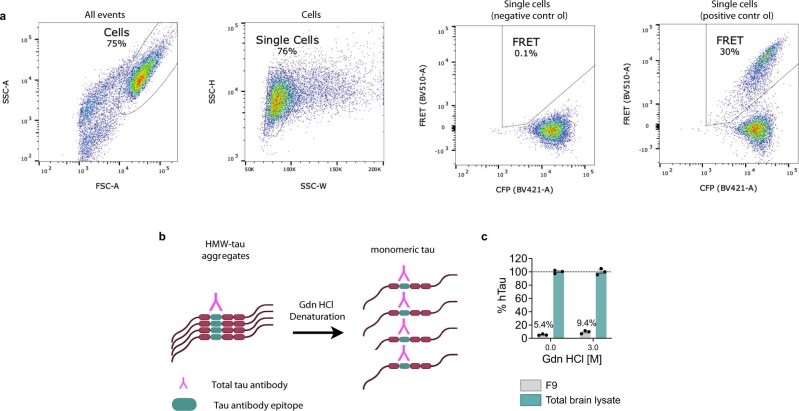
HMW tau seed represents a low percentage of total tau in the PS19 mouse brain. **a**, Cells were gated on a FSC-A vs SSC-A plot. From the gated cells population, single cells were gated on a SSC-W vs SSC-H plot. From the gated single cells population, FRET positive cells were gated on a BV421-A vs BV510-A plot after compensation. The FRET-positive population lies in the region with the same BV421-A intensity, but higher BV510-A intensity, with the boundary between positive and negative determined by excluding the signal from a negative control. **b**, Schematic of aggregated tau denaturation by guanidine HCl (Gdn HCl). **c**, hTau present in F9 (gray bars) as a percentage of total brain lysates (green bars) before and after denaturation with 3 M of Gdn-HCl. Total human tau levels were assayed by ELISA. Data represent the mean ± s.e.m. The experiment was performed with *n* = 3. Source data

We characterized tau seeds from the PS19 mouse model that overexpresses human tau harboring the p.Pro301Ser (P301S) mutation^23^. To assess the molecular weight of the tau species involved in seeding activity, we performed size exclusion chromatography (SEC) on Tris-buffered saline (TBS)-soluble brain extracts from 3-month-old PS19 mice, representing an early stage of pathology. The tau-seeding activity of each SEC fraction was assessed by transfection into tau RD P301S fluorescence resonance energy transfer (FRET) biosensor cells and quantification of the integrated FRET density by flow cytometry^24^ (Fig. 1a and Extended Data Fig. 1a). The strongest seeding activity was found in the void volume fraction, fraction 9, which contained HMW proteins larger than 2,000 kDa (Fig. 1b). Fraction 9 contained only 5% of total tau, as assayed by enzyme-linked immunosorbent assay (ELISA) for human tau (Fig. 1c and Extended Data Fig. 1b,c). Interestingly, fraction 9 contained a low percentage of total tau even after treatment with the chaotropic denaturant guanidine hydrochloride (Gdn-HCl), which unmasks hidden epitopes^25,26^ (Extended Data Fig. 1b,c), meaning that the low total tau level detected in fraction 9 is not due to epitope masking in the HMW tau complex (Extended Data Fig. 1b,c). No seeding activity was observed in any SEC fractions from wild-type (WT) littermates (Fig. 1b).

To confirm that the tau species present in fraction 9 is responsible for the seeding activity, we depleted human tau via immunoprecipitation (IP) using the HT7 antibody and observed a dramatic decrease in the seeding activity of the flow-through (Fig. 1d). Electron microscopy (EM) of the tau IP material from fraction 9 revealed that the seed takes on predominantly short filaments structure with a width of 7 ± 3 nm (Fig. 1e).

We performed IP of human tau from fraction 9 and fraction 17 (containing monomeric tau) using the HT7 antibody to identify protein interactors of the HMW tau seed and monomeric tau isolated from the same brain. The IP products were analyzed by a TMT-tags-based quantitative mass spectrometry workflow (Fig. 1f,g and Supplementary Data 1). DAVID functional annotation clustering revealed enrichment of synaptic terms in both interactomes (Supplementary Data 2). Interestingly, many synaptic proteins identified as tau-seed interactors differed from those that interacted with monomeric tau (Supplementary Data 2). We compared both interactomes with potential therapeutic targets for AD nominated by the AMP-AD in the ‘Wall of Targets’ (Supplementary Data 2)^27,28^. From this comparison, we identified BSN, a scaffolding protein of the presynaptic active zone involved in regulating synaptic neurotransmitter release^22^, presynaptic proteostasis and autophagy^29–31^, as a significant interactor of the tau seed. Surprisingly, missense mutations in the *BSN* gene have recently been identified in a family with pathological aggregation of 3R/4R tau^32^. Additionally, BSN expression is increased in patients with multiple system atrophy^33^ and BSN accumulates in patients with multiple sclerosis^34^.

**Extended Data Fig. 2:**
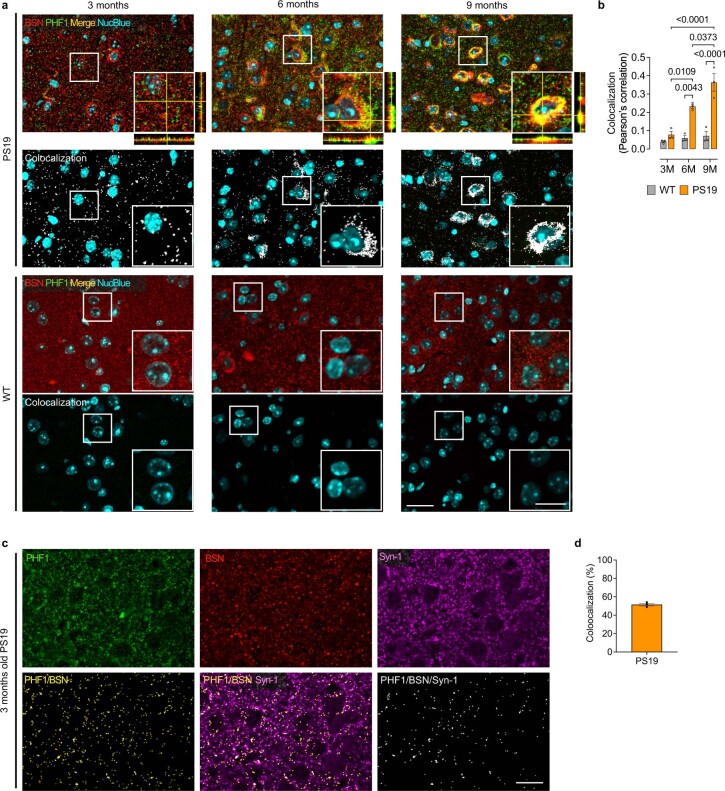
Bassoon is associated with tau pathology in PS19 mice. **a**, Representative immunofluorescence and colocalization for BSN (red), PHF1 (green), nuclei (cyan), and merge (yellow), in 3-, 6- and 9-month-old wild-type and PS19 mouse cortexes. Scale bar: 200 µm for main images and 50 µm for insets. Merge image for PS19 includes orthogonal image of reconstructed three-dimensional views. Colocalization analysis was performed to determine pixel intensity correlation between PHF1 and BSN. **b**, Pearson’s correlation of BSN and PHF1 intensity in 3-, 6- and 9-month-old wild-type and PS19 mouse cortexes. **c**, 3-month-old PS19 mouse sections immunostained against PHF1, BSN, and Syn-1 (upper panels), and colocalization of PHF1 and BSN (colored yellow; lower left panel). PHF1/BSN colocalization was re-colocalized with the Syn-1 channel to obtain a final triple-colocalization between PHF1/BSN and Syn-1 (lower middle and right panels). **d**, Percentage of colocalization between PHF1/BSN puncta and Syn-1. Data are the mean ± s.e.m. Experiments were performed with *n* = 3 (**a**–**d**). Significance was determined by one-way ANOVA (**b**). Source data

We confirmed the interaction between BSN and tau seeds by co-immunoprecipitation (co-IP) in the SEC fraction 9 from PS19 mice (Fig. 1h). Double staining also revealed a strong colocalization of BSN with tau deposits in PS19 mice (Fig. 1i and Extended Data Fig. 2). The degree of colocalization between BSN and phosphorylated tau increased with age (Extended Data Fig. 2a,b). At later stages of tau pathology (9 months old), the colocalization of both signals appeared to be cytoplasmic, but at early stages (3 months old), the colocalization presented as diffuse puncta. Considering that BSN is mainly a presynaptic protein and tau has been identified at the presynapse at the early stage of pathology^35,36^, we performed triple staining for BSN, phospho-tau PHF1, and the presynaptic marker synapsin 1 (Syn-1) at 3 months, to show that BSN and phospho-tau colocalize at the presynaptic terminals (Extended Data Fig. 2c,d). These results suggest that at the early stages of pathology, the tau seed interacts with BSN at presynaptic compartments, and as the disease progresses, tau aggregates and BSN co-deposit in the cytoplasm.

### BSN is associated with tau pathology in human tauopathy brain tissue

**Fig. 2:**
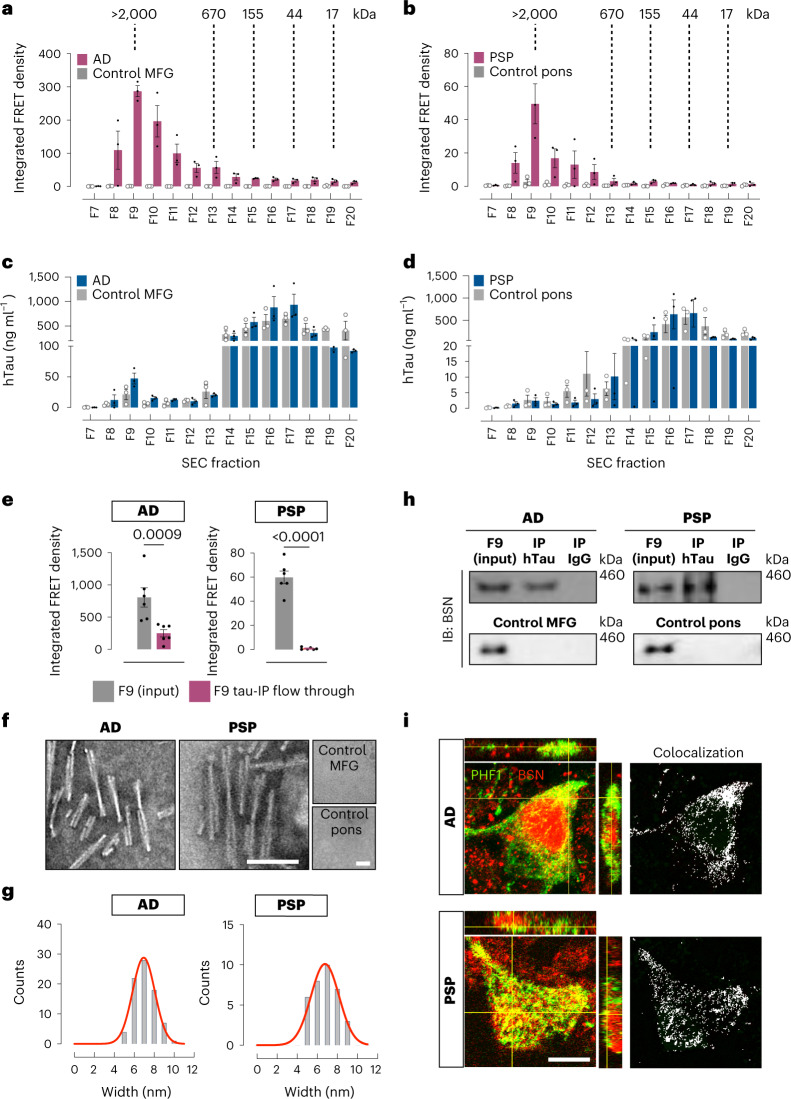
Bassoon is associated with tau pathology in human Alzheimer’s disease and progressive supranuclear palsy. **a**,**b**, Tau-seeding activity of SEC fractions from human AD lysates (from middle frontal gyrus, MFG; **a**) and PSP lysates (from pons; **b**), compared to healthy controls from MFG and pons, respectively. **c**,**d**, Total human tau detected by ELISA in SEC fractions from human AD (**c**) and PSP (**d**) brain lysates compared to healthy controls. **e**, Tau-seeding activity of the AD and PSP SEC fraction 9 (F9) containing HMW tau, before and after hTau IP using HT7 antibody. **f**,**g**, Tau twisted filaments present in the tau-IP product from F9 of AD and PSP SEC fractions, visualized by EM (scale bars, 100 nm; **f**), and width distribution (**g**). No filaments were detected in MFG and pons healthy controls. **h**, Co-IP of human tau (HT7) and BSN from SEC F9 in AD, PSP, control MFG and control pons brain lysates. **i**, Colocalization (yellow/white) between BSN (red) and pathological phosphorylated tau species (PHF1, green) in AD and PSP brain sections. The merged panel includes orthogonal images of reconstructed three-dimensional views. Colocalization analysis was performed to determine pixel intensity correlation between PHF1 and BSN. Scale bar, 10 µm. Data are shown as the mean ± s.e.m. Experiments were performed with *n* = 3 (**a**–**d**, **f**, **h** and **i**) and *n* = 6 (**e**). Significance was determined by unpaired two-tailed Student’s *t*-test (**e**). Source data

**Extended Data Fig. 3:**
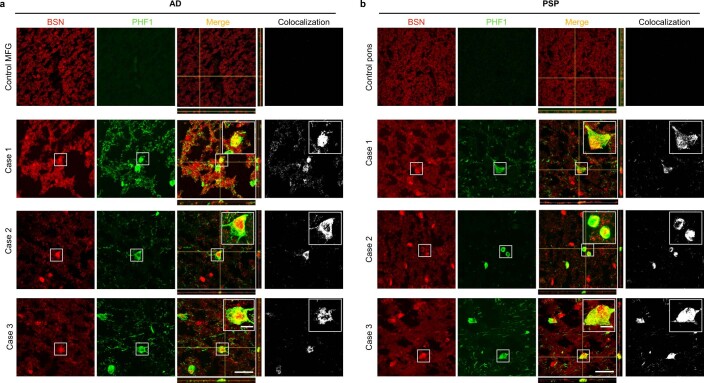
Bassoon colocalizes with a pathological tau species in human AD and PSP cases. **a, b**, Immunofluorescence, merge and colocalization images of BSN (red) and pTauS396/S404 (PHF1, green) in human AD (from middle frontal gyrus, MFG) (**a**), and PSP (from pons) (**b**), and healthy control subjects from MFG and pons, respectively. Merge image includes orthogonal image of reconstructed three-dimensional views. Scale bar: 200 µm for main images and 50 µm for insets. Experiments were performed in triplicates for AD and PSP cases.

The tau species with the strongest seeding activity in AD and PSP patient brains was also a HMW tau species in fraction 9 (>2,000 kDa; Fig. 2a,b). As in the PS19 model, for both human tauopathies, tau in fraction 9 represented a small percentage of total tau in the brain (Fig. 2c,d). Interestingly, the age-matched controls had similar levels of tau in fraction 9 but lacked seeding activity (Fig. 2a–d). Depleting tau in fraction 9 from AD and PSP brains dramatically decreased seeding activity in the flow-through (Fig. 2e). EM of the tau IP material from fraction 9 revealed that in AD and PSP, the seeds were predominantly twisted filaments with widths of 6.95 ± 1.1 nm and 6.77 ± 1.4 nm, respectively (Fig. 2f,g). No tau filaments were detected when tau was immunoprecipitated from age-matched controls (Fig. 2f). We then confirmed by co-IP that, in both tauopathies, BSN interacts with tau in fraction 9 but does not interact with tau in fraction 9 from age-matched controls (Fig. 2h). Double staining revealed colocalization of BSN with tau deposits in AD and PSP brains (Fig. 2i and Extended Data Fig. 3).

### BSN enhances tau-seeding activity and toxicity

**Fig. 3:**
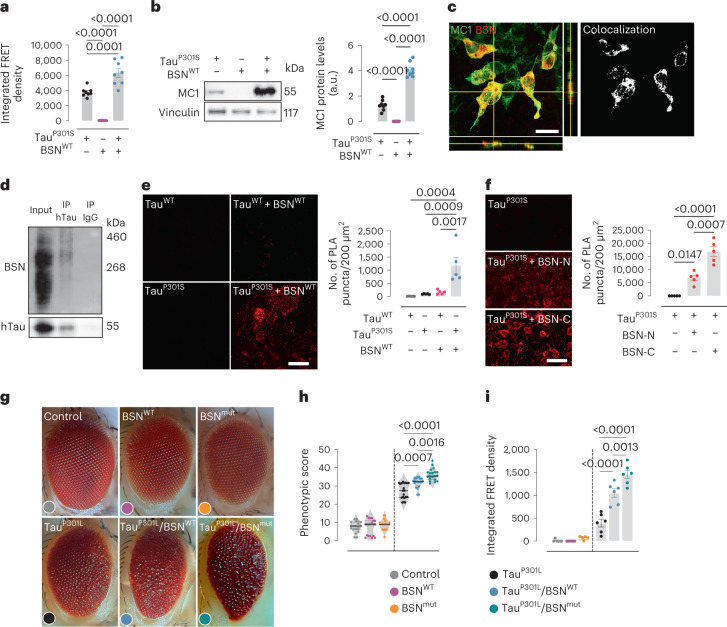
Bassoon overexpression increases tau seeding and toxicity by direct interaction. **a**, Seeding activity of HEK cell lysates expressing hTau^P301S^, BSN^WT^ or both plasmids. **b**, Western blot and quantification of misfolded tau levels detected with MC1 antibody in HEK cells overexpressing hTau^P301S^, BSN^WT^ or both. **c**, Representative image of double immunofluorescences between MC1 and BSN in HEK cells overexpressing hTau^P301S^ and BSN^WT^. The merged panel includes orthogonal images of reconstructed three-dimensional views. Colocalization analysis was performed to determine pixel intensity correlation between MC1 and BSN. Scale bar, 50 µm. **d**, Co-IP of hTau (HT7 antibody) and immunoblot for BSN in HEK cells overexpressing hTau^P301S^ and BSN^WT^. **e**, PLA fluorescence pairing of hTau and BSN antibodies, and quantification in HEK cells overexpressing Tau^WT^, Tau^WT^/BSN^WT^, Tau^P301S^ and Tau^P301S^/BSN^WT^. **f**, PLA fluorescence pairing of hTau and His-tag antibodies, and quantification in HEK cells overexpressing Tau^P301S^ and Tau^P301S^ together with the N-terminal or C-terminal fragments of BSN^WT^ (BSN-N or BSN-C, respectively). Scale bar, 50 µm. **g**–**i**, Representative images (**g**), quantification of phenotypic eye degeneration (**h**) and tau-seeding activity (each point is a pool of 20 fly heads from a distinct eclosion event; **i**), in control, BSN^WT^, BSN^mut^, hTau^P301L^, hTau^P301L^/BSN^WT^ and hTau^P301L^/BSN^mut^ flies. Data are the mean ± s.e.m. Experiments were performed with *n* = 8 (**a**–**c**), *n* = 3 (**d**), *n* = 5 (**e** and **f**), *n* = 20 (**g** and **h**) and *n* = 6 (**i**). Significance was determined by one-way analysis of variance (ANOVA; **a**, **b**, **e**, **f**, **h** and **i**). a.u., arbitrary units. Source data

**Extended Data Fig. 4:**
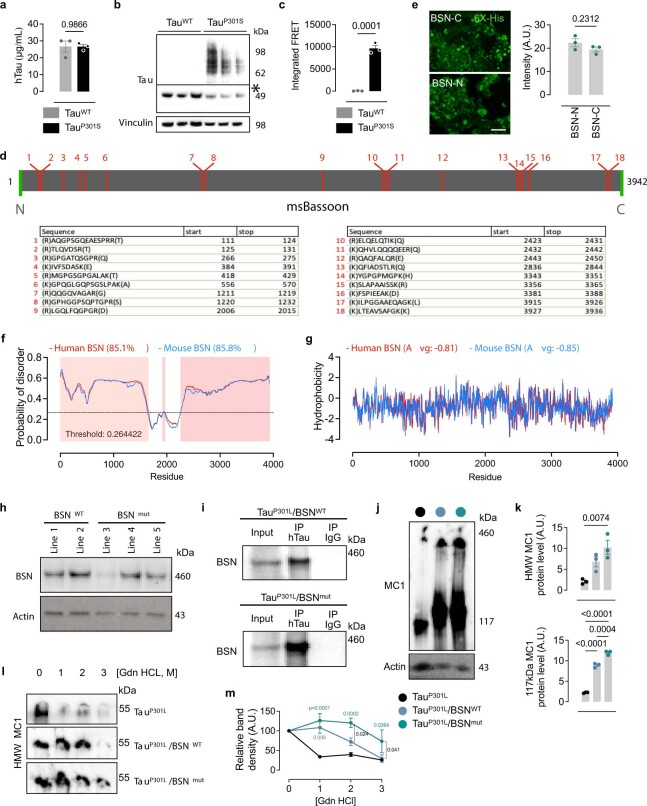
Bassoon overexpression increases tau accumulation *in vitro* and in a *Drosophila* model of tauopathy. **a**–**c**, hTau levels by ELISA (**a**), Western blot of total tau (**b**), seeding activity (**c**) from HEK cells overexpressing hTau^WT^ or hTau^P301S^. Western blot membrane was overexposed in the upper level for better visualization of oligomeric tau (denoted by the asterisk*). **d**, Location of BSN peptides detected by IP^−^mass spec. **e**, Representative images, and quantification of 6X-His immunofluorescences from HEK cells overexpressing hTau^P301S^ and 6X-His-BSN N- or C- terminal fragments (BSN-N or BSN-C, respectively). **f**, Disordered residues of human and mouse bassoon. 85.1% of residues in human BSN and 85.8% of residues in mouse BSN were predicted to be disordered. **g**, Hydrophobicity profile of bassoon using method of Kyte & Doolittle. Average hydrophobicity is −0.81 for human BSN, and −0.85 for mouse BSN**, h**, Western blot confirming the overexpression of the UAS-BSN under the GMR-Gal4 driver in different fly lines. **i**, Western blot for BSN of co-immunoprecipitation (Co-IP) of human tau (HT7) from hTau^P301L^/BSN^WT^ and hTau^P301L^/BSN^mut^ fly head lysates. Experiments were repeated three times with similar results (**h**, **i**). **j**, **k**, Western blot (**j**), of the level of HMW and 117 kDa MC1 detected and quantified (**k**), in hTau^P301L^, hTau^P301L^/BSN^WT^ and hTau^P301L^/BSN^mut^ fly head lysates. Each sample is a pool of 20 fly brains from 3 different eclosion events. **l**, **m** Western blot (**l**), and quantification of misfolded tau (MC1) (**m**), in fly lysates denatured by increasing concentrations of guanidine HCl, in hTau^P301L^, hTau^P301L^/BSN^WT^, and hTau^P301L^/BSN^mut^ head lysates. Data are the mean ± s.e.m. Experiments were performed with *n* = 3 (**a**–**c**, **e**, **j**–**m**). Significance was determined by unpaired two-tailed Student’s t-test (**a**, c, **e**) and one-way ANOVA (**k**, **m**). Source data

We overexpressed human P301S tau in human embryonic kidney (HEK) 293T cells with and without human BSN to determine the effect of BSN on the pathological properties of tau. Lysates from cells overexpressing P301S tau with BSN demonstrated increased seeding activity compared to the lysate from cells overexpressing solely P301S tau (Fig. 3a). The overexpression of BSN was also associated with an increased accumulation of misfolded tau species (Fig. 3b). Double staining of HEK cells coexpressing BSN and P301S tau demonstrated that BSN co-deposits with misfolded tau species (Fig. 3c). In the same coexpression experiment, we confirmed by co-IP that BSN interacts with P301S tau (Fig. 3d). We performed a proximity ligation assay (PLA) on HEK cells to determine whether BSN interacts directly with tau. Cells coexpressing P301S tau and BSN showed a strong PLA signal, indicating that P301S tau and BSN are within interaction proximity (<40 nm; Fig. 3e). Considering that BSN interacts with the tau seed but not monomeric tau from PS19 mice (Fig. 1f,g) and with fraction 9 tau from patients with AD and PSP but not that from age-matched controls without seeding activity (Fig. 2h), we aimed to determine whether this interaction was conformation dependent. Because overexpressing a similar amount of human WT tau did not form stable aggregates and did not show the same seeding activity as human P301S tau (Extended Data Fig. 4a–c), we performed PLA on HEK cells coexpressing BSN and human WT tau to determine if BSN interacts with non-aggregating tau. Minimal PLA signal was observed in cells overexpressing WT tau and BSN (Fig. 3e), demonstrating that BSN has a higher affinity for tau when tau adopts a misfolded or aggregated conformation.

In the tau-seed analysis comprising immunoprecipitation followed by mass spectrometry (IP–MS; Fig. 1f), we identified BSN peptides corresponding to the N-terminal and the C-terminal regions of the BSN protein (Extended Data Fig. 4d), suggesting that the tau seed interacts with full-length BSN. We coexpressed P301S tau with the N-terminal fragment (1–850) or with the C-terminal fragment (2,450–3,942) of human BSN fused to a 6x-His tag in HEK cells, with both fragments expressed at a similar level (Extended Data Fig. 4e), to determine the region of BSN that interacts with aggregated tau. We then performed PLA and observed that cells co-transfected with P301S tau and BSN C-terminal fragment had a strong PLA signal (Fig. 3f), suggesting that BSN could interact with tau aggregates through its C-terminal region. Nevertheless, although significantly lower, PLA signals were observed in HEK cells co-transfected with P301S tau and BSN N-terminal fragment (Fig. 3f), suggesting that BSN could also interact with tau aggregates via its N-terminal region. Interestingly, it has been shown that large proteins with lower-than-average hydrophobicity, more intrinsically disordered residues and longer regions of disorder are more susceptible to aberrant interactions with amyloid-like aggregates^37^. Considering that BSN is a large protein that is almost entirely disordered^34^ (Extended Data Fig. 4f) and has a lower-than-average hydrophobicity (Extended Data Fig. 4g), BSN has the potential to engage in widespread aberrant interactions with aggregated forms of tau.

We overexpressed human BSN in two transgenic *Drosophila melanogaster* lines to investigate the effects of BSN upregulation in vivo, one line overexpressing WT BSN and the other overexpressing BSN harboring the p.Pro3866Ala mutation, recently identified in patients with pathological aggregation of 3R/4R tau^32^ (Extended Data Fig. 4h). Neither of these lines developed a degenerative eye phenotype (Fig. 3g). However, the overexpression of WT BSN enhanced the degenerative eye phenotype in the *Drosophila* model expressing human tau with the p.pro301leu mutation (hTau-P301L) by increasing the disruption of the ommatidial structure (Fig. 3g,h). The degenerative eye phenotype was intensified in the hTau-P301L fly when the BSN mutant was overexpressed (Fig. 3g,h). We confirmed by co-IP that WT and mutant BSN also interacted with tau in flies (Extended Data Fig. 4i). Western blotting showed that both WT and mutant BSN promoted the accumulation of the misfolded tau species, as detected by MC1 antibody (Extended Data Fig. 4j,k). We then used a guanidine stability assay to further examine stability differences between fly hTau-P301L aggregates. In the presence of WT and mutant BSN, tau aggregates were significantly more resistant to disaggregation by Gdn-HCl (Extended Data Fig. 4l,m). As observed in our cellular model, BSN overexpression in hTau-P301L flies led to an increase in tau-seeding activity, which was even higher with mutant BSN (Fig. 3i).

### *Bsn* knockdown reduces tau spread in vivo

**Fig. 4:**
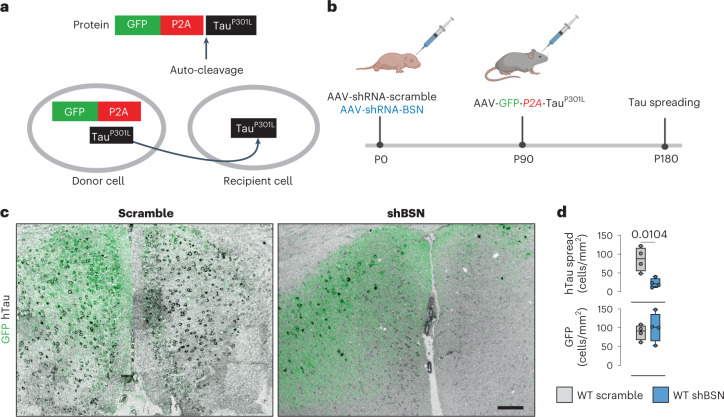
Bassoon contributes to tau spreading in vivo. **a**, Schematic of the GFP-P2A-hTau^P301L^ tau-spreading mouse model. GFP^+^ neurons are hTau donors, spreading hTau^P301L^ to recipient GFP^−^ neurons. **b**, Timeline of injections in WT mice. Animals were injected with scramble or shBSN AAV at P0, then with AAV-GFP-P2A-hTau^P301L^ at P90. Animals were euthanized at P180 to evaluate hTau spreading. **c**, Representative immunofluorescences for hTau (HT7 antibody) in animals overexpressing GFP-P2A-hTau^P301L^ injected with scramble shRNA and BSN shRNA. Scale bar, 100 µm. **d**, Quantification of cortical hTau^+^/GFP^−^ cells (top) and GFP^+^ cells (bottom) per mm^2^. For box plots, the center line represents the median, boundaries denote the interquartile range, and the whiskers represent the lower and upper limits. Values are given as the means ± s.e.m. Experiments were performed with *n* = 4, and significance was determined by unpaired two-tailed Student’s *t*-test. Source data

**Extended Data Fig. 5:**
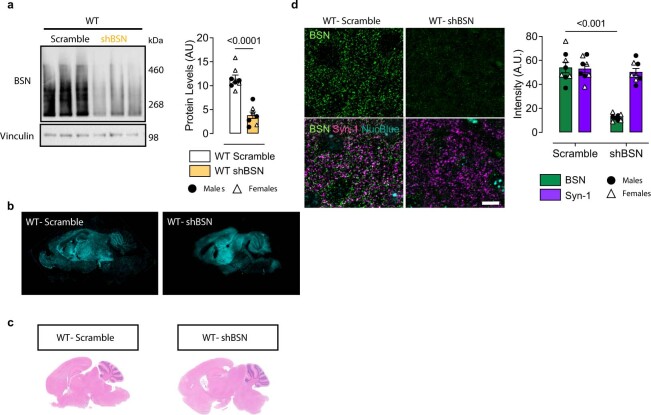
Murine bassoon downregulation does not produce gross brain abnormalities. **a**, Western blot and quantification of BSN downregulation in WT mice. **b**, Detection of BFP2 reporter in AAV scramble and AAV shBSN mice, confirming the widespread expression of both sequences. **c**, H&E staining of WT mice injected with a scramble and shBSN shRNA. **d**, BSN and Syn-1 immunofluorescence in WT mouse cortexes injected scramble and shBSN shRNA, and mean intensity of BSN and Syn-1. Data are shown as the mean ± s.e.m. Experiments were performed with *n* = 8 (**a**–**d**). Significance was determined by unpaired two-tailed Student’s t-test (**a**), and one-way ANOVA (**d**). Source data

We then aimed to investigate whether BSN is critical for tau spreading in the brain using a well-characterized adeno-associated virus (AAV)-based spreading model. In this model, GFP and hTau^P301L^ proteins are translated from mRNA-GFP-P2A-hTau^P301L^ under the control of the cytomegalovirus promoter^38^. This model allows for discrimination between transduced neurons expressing both GFP and hTau^P301L^ from those expressing only hTau^P301L^ due to spreading^38^ (Fig. 4a). We performed neonatal (postnatal day (P) 0) intracerebroventricular injection of an AAV harboring a short-hairpin RNA (shRNA) against mouse *Bsn* (shBSN) or control ‘scramble’ shRNA in WT mice to downregulate the expression of *Bsn* in parallel. shBSN significantly downregulated *Bsn* (to ~60%) expression in vivo in WT mice (Extended Data Fig. 5a). Both scramble and shBSN shRNAs encode a blue fluorescent protein (BFP2) reporter, allowing the visualization of AAV transduction throughout the brain without producing gross abnormalities or negatively affecting presynaptic integrity (Extended Data Fig. 5b–d). Specifically, neonatal (P0) mice received intracerebroventricular injections of pAAV9-mTagBFP2-U6-mBsn-shRNA or control pAAV9-mTagBFP2-U6-Scr-shRNA, and, 3 months later, we administered stereotaxic injections of the pAAV-GFP-(P2A)-hTau^P301L^ into the hippocampus. Three months after injection, the mice were euthanized, and tau spread was assessed by immunostaining using the anti-hTau HT7 antibody (Fig. 4b,c). Tau spreading was quantified in both groups by counting the number of hTau^+^/GFP^−^ cells per mm^2^ (Fig. 4c,d). We observed substantial tau spreading in mice injected with scramble AAV, whereas spreading was significantly reduced in *Bsn* knockdown mice (Fig. 4d). This decrease was not due to differences in the number of transduced cells, as a comparable number of GFP-positive cells was observed in both groups (Fig. 4d).

### *Bsn* downregulation reduced pathology and seed stability in vivo

**Fig. 5:**
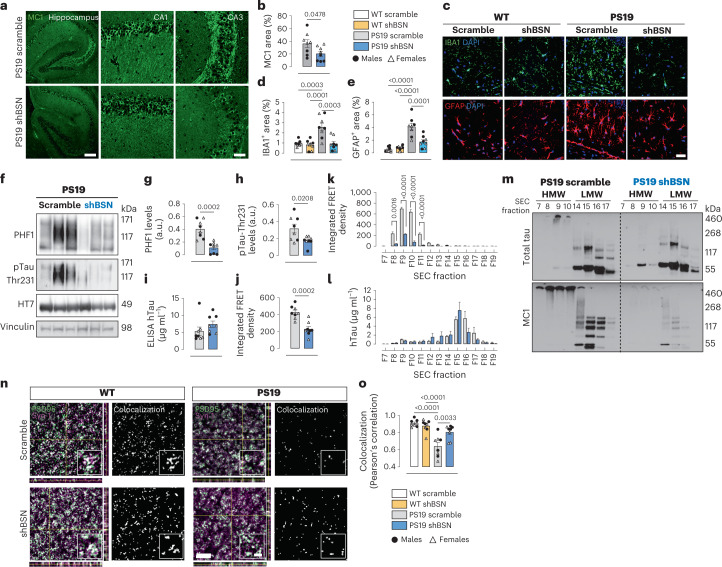
Bassoon downregulation reduces tau pathology and tau-seeding stability in vivo. **a**, Representative images of hippocampal MC1 immunostaining in 4-month-old PS19 mice injected with shBSN and scramble shRNA. Scale bars, 200 µm and 50 µm for CA1 and CA3 insets, respectively. **b**, Quantification of MC1 immunostainings in shBSN and scramble PS19 mice as a percentage of area. **c**–**e**, Hippocampal immunofluorescence (**c**), using specific antibodies against GFAP (red) and IBA1 (green) in WT_scramble_, WT_shBSN_, PS19_scramble_ and PS19_shBSN_ mice. Quantification was performed as a percentage of area for IBA1^+^ (**d**) and GFAP^+^ (**e**) cells. **f**–**h**, Western blot (**f**), and quantification of specific antibodies against pTau-Ser396/Ser404 (PHF1; **g**), and pTau-Thr231 (**h**), in shBSN and scramble PS19 mouse brain. **i**,**j**, Total human tau levels measured by ELISA (**i**), and tau-seeding activity (**j**), in shBSN and scrambled shRNA PS19 mouse brain lysates. **k**,**l**, Seeding activity (**k**) and total hTau levels (**l**) in SEC fractions from shBSN or scramble PS19 mouse brain lysates. **m**, Western blot of total tau (HT7, top blot) and misfolded tau (MC1, bottom blot) in SEC fractions (fractions 7 to 10, HMW; fractions 14 to 17, low molecular weight (LMW)) from shBSN and scramble PS19 mouse brain lysates. The experiment was repeated three times with similar results. **n**, Representative merge images of immunofluorescence of PSD95 (green), Syn-1 (magenta) and colocalized pixels (white). The merged panel includes orthogonal images of reconstructed three-dimensional views. Colocalization analysis was performed to determine pixel intensity correlation between PSD95 and Syn-1. **o**, Colocalization quantification by Pearson’s correlation coefficient. Data are the mean ± s.e.m. Experiments were performed with *n* = 8 (**a**–**j**, **n** and **o**), *n* = 6 (**k** and **l**) and *n* = 3 (**m**). Significance was determined by unpaired two-tailed Student’s *t*-test (**b** and **g**–**k**) and one-way ANOVA (**d**, **e** and **o**). Source data

**Extended Data Fig. 6:**
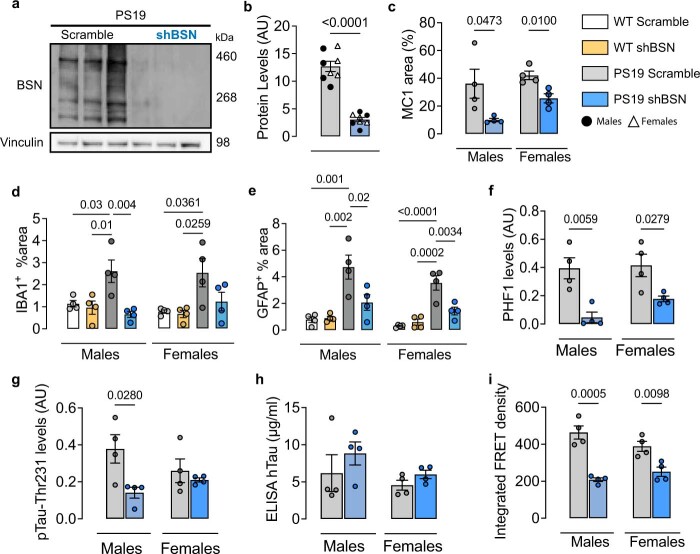
Reducing bassoon levels mitigate tau pathology in male and female PS19 mice. **a**, **b**, Western blot (**a**), and quantification of BSN downregulation (**b**), in PS19 mice. **c**, Quantification of MC1 immunostaining in shBSN and scramble, of PS19 mice by sex as a percentage of area. **d**, **e**, Quantification of hippocampal immunofluorescence of IBA1 (**d**), and GFAP (**e**), in WT_scramble_, WT_shBSN_, PS19_scramble,_ and PS19_shBSN_ mice, separated by sex. **f**, **g**, Western blot quantification of pTauS396/S404 (PHF1) (**f**), and pTau Thr231 (**g**), in shBSN and scramble PS19 mouse brain lysate, separate by sex. **h**, **i**, Total human tau levels by ELISA (**h**), and tau-seeding activity (**i**), in shBSN and scrambled PS19 mouse brain lysates, separated by sex. Data are shown as the mean ± s.e.m. Experiments were performed with *n* = 8 (**a**, **b**), and *n* = 4 per sex (**c**–**i**). Significance was determined by unpaired two-tailed Student’s t-test (**b, c, f**–**i**), and one-way ANOVA (**d**, **e**). Source data

**Extended Data Fig. 7:**
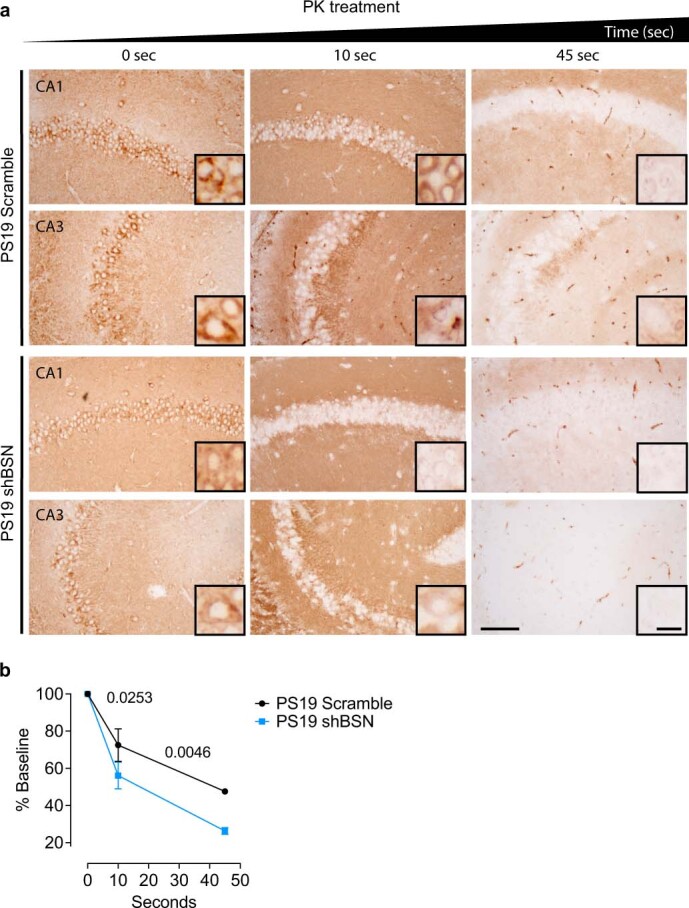
Increased susceptibility to PK degradation of misfolded tau aggregates after bassoon downregulation in PS19 mice. **a**, **b**, Proteinase K sensitivity assay (**a**), and quantification (**b**), of MC1 IHC intensity at 0, 10, and 45 sec after PK treatment in scramble and shBSN PS19 brain sections. Scale bar: 100 µm for main pictures and 10 µ for insets. Data are shown as the mean ± s.e.m. Experiments were performed with *n* = 3 (**a**, **b**). Significance was determined by unpaired two-tailed Student’s t-test (**b**). Source data

We next investigated whether *Bsn* downregulation affects tau pathology and associated pathogenesis in PS19 mice. Neonatal PS19 and WT littermates were injected with an AAV encoding scramble or *Bsn* shRNA. Four months after injection, the mice were euthanized, and western blotting was conducted to confirm *Bsn* downregulation (Extended Data Fig. 6a,b). Detection of the anti-misfolded tau antibody (MC1) revealed that decreasing BSN levels reduced tau pathology in the hippocampus of PS19 mice (Fig. 5a,b). Microgliosis and astrogliosis were similarly decreased (Fig. 5c–e). The effect of *Bsn* downregulation on tau pathology and gliosis was significant in both female and male PS19 mice (Extended Data Fig. 6c–e). Western blotting in non-reducing conditions revealed that *Bsn* downregulation decreased phosphorylated tau aggregates (pTau; detected using PHF1 (pSer396/Ser404) and pThr231 antibodies) in the total lysate (Fig. 5f–h). ELISA revealed that *Bsn* downregulation did not influence total human tau levels in PS19 brains (Fig. 5i); however, a dramatic decrease in tau-seeding activity was observed (Fig. 5j). *Bsn* downregulation significantly decreased the levels of PHF1 aggregates, and seeding activity in females and males, but the effect on pThr231 tau levels was solely observed in male PS19 mice (Extended Data Fig. 6f–i). We then performed SEC on the brain lysates to determine if *Bsn* downregulation affects HMW tau-seed activity. As expected, the seeding activity of the fraction containing the HMW tau seed (fraction 9) was significantly lower in PS19_shBSN_ than in PS19_scramble_ samples (Fig. 5k). Interestingly, a similar distribution of total tau levels was observed in the SEC fractions of PS19_shBSN_ and PS19_scramble_ mice (Fig. 5l), suggesting that BSN plays a minimal role in the formation of HMW tau complexes but instead affects the properties of the tau seed by enhancing its seeding activity, perhaps by stabilizing and promoting HMW tau-seed aggregation. SEC fractions were analyzed by western blot with strong reducing conditions to test this hypothesis. Tau remained at the top of the gel in the HMW fractions (F8, F9 and F10) in samples from PS19_scramble_, but in samples from PS19_shBSN_, HMW tau was resolved in the gel as a monomer (Fig. 5m). When using the MC1 antibody, tau again remained at the top of the gel in HMW SEC fractions in PS19_scramble_ samples, but in PS19_shBSN_ samples, no misfolded tau was observed in the HMW SEC fractions in strong reducing conditions (Fig. 5m). These data suggest that BSN stabilizes HMW tau aggregates, increasing their resistance to degradation in reducing conditions. We assessed the proteinase K (PK) sensitivity of misfolded tau in brain sections from PS19_scramble_ and PS19_shBSN_ mice to further explore if BSN affects the structural properties of tau aggregates. Misfolded tau detected by MC1 in PS19_shBSN_ samples was considerably more sensitive to protease degradation, with the intensity dramatically decreased after 45 s of PK digestion (Extended Data Fig. 7). This result suggests that in the absence of BSN, tau aggregates have a relaxed structure and are accessible for digestion, whereas in the presence of BSN, tau aggregates are more compacted and resistant to PK.

**Extended Data Fig. 8:**
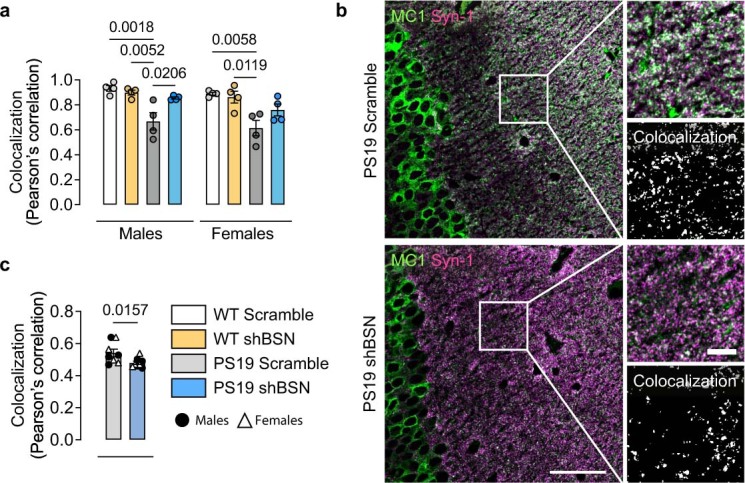
Rescue of synaptic integrity in PS19 after bassoon downregulation. **a**, Pearson’s correlation of PSD95 and Syn-1 in WT_scramble_, WT_shBSN_, PS19_scramble,_ and PS19_shBSN_ mice, separated by sex. **b**, Representative immunofluorescence in scramble and shBSN PS19 mice for misfolded tau (MC1), and Syn-1 protein. Colocalization analysis was performed to determine pixel intensity correlation between MC1 and Syn-1. Scale bar: 50 µm for main panels and 10 µm for insets. **c**, Pearson’s correlation of MC1 and Syn-1 immunostainings in scramble and shBSN PS19 mice. Data are shown as the mean ± s.e.m. Experiments were performed with *n* = 4 per sex (**a**), and *n* = 8 (**b, c**). Significance was determined by one-way ANOVA (**a**), and unpaired two-tailed Student’s t-test (**c**). Source data

Because synaptic loss occurs as early as 3 months of age in PS19 mice^23^, we assessed whether the decrease in tau pathology after *Bsn* downregulation corresponded to improved synaptic integrity in 4-month-old PS19 mice. Quantification of the colocalization of presynaptic (Syn-1) and postsynaptic (PSD95) marker puncta in the cortex of PS19_scramble_ and PS19_shBSN_ mice (Fig. 5n) revealed a significant improvement in synaptic integrity in PS19_shBSN_ mice (Fig. 5o and Extended Data Fig. 8a). The effect of *Bsn* downregulation on synaptic integrity in PS19 mice could be due to a decrease in the accumulation of misfolded tau at the presynapse (Extended Data Fig. 8b,c).

### Reducing BSN rescues the phenotypes in a tauopathy mouse model

**Fig. 6:**
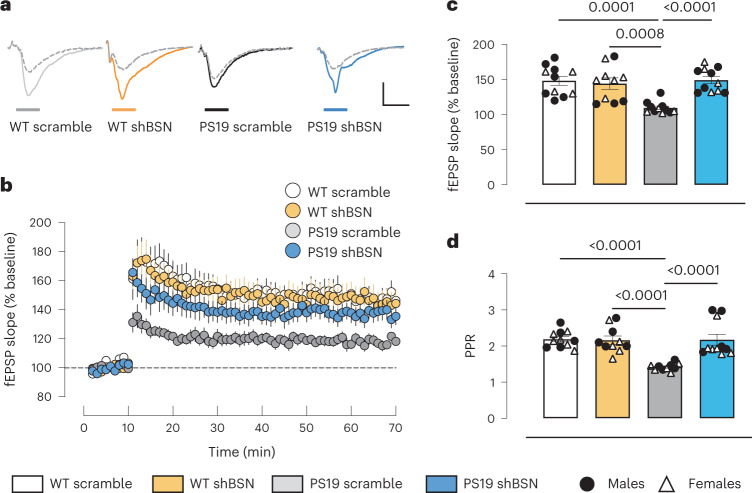
Bassoon downregulation restores electrophysiological impairments in PS19 mice. **a**, Superimposed fEPSP traces produced by stimulation of the Schaffer collateral pathway and recorded in CA1 brain slices before (gray dashed lines) and after (gray, orange, black and light blue lines) LTP induction. Vertical bar, 300 mV; horizontal bar, 10 ms. **b**–**d**, Summary of in vivo LTP (**b**), averaged from minute 60 to 70 (**c**), and PPR (**d**), in 6-month-old WT and PS19 mice injected with shBSN or scramble shRNA. Data represent the mean ± s.e.m. Experiments were performed with *n* = 11 for WT_scramble_, *n* = 11 for PS19_scramble_, *n* = 10 for WT_shBSN_ and *n* = 11 for PS19_shBSN_ mice (**b**–**d**). Significance was determined by two-way ANOVA (**c** and **d**). Source data

**Extended Data Fig. 9:**
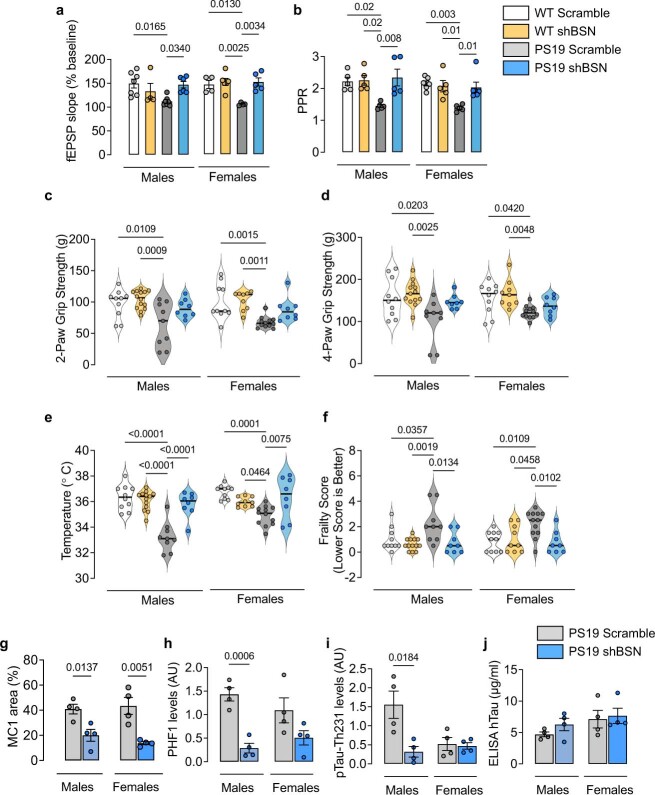
Bassoon downregulation improves behavioral and electrophysiological impairments in PS19 males and females. **a**, **b**, Average of fEPSP slope from minute 60 to 70 by sex (**a**) and paired-pulse ratio (PPR) (**b**), in 6-month-old WT and PS19 mice injected with shBSN or scramble shRNA. **c**-**f**, 2-paw test (**c**), 4-paw test (**d**), body temperature (**e**), and frailty test (**f**), in 9-month-old WT and PS19 mice injected with shBSN or scramble shRNA. **g**-**j**, Quantification of MC1 immunostaining as a percentage of area (**g**), pTauS396/S404 (PHF1) protein levels (**h**), pTau Thr231 protein levels (**i**), and total human tau levels by ELISA (**j**), in shBSN and scrambled PS19 male and female mouse brain lysates. Data represent the mean ± s.e.m. Experiments were performed with *n* = 7 for males and *n* = 4 for females for WT_scramble_, *n* = 4 for males and *n* = 6 for females for PS19_scramble_, *n* = 7 for males and *n* = 4 for females for WT_shBSN_, and *n* = 6 for males and *n* = 5 for females for PS19_shBSN_ mice (**a**), *n* = 5 for males and *n* = 6 for females for WT_scramble_, *n* = 5 for males and *n* = 5 for females for PS19_scramble_, *n* = 5 for males and *n* = 5 for females for WT_shBSN_, and *n* = 5 for males and *n* = 6 for females for PS19_shBSN_ mice (**b**), *n* = 10 for males and *n* = 10 for females for WT_scramble_, *n* = 9 for males and *n* = 12 for females for PS19_scramble_, *n* = 13 for males and *n* = 9 for females for WT_shBSN_, and *n* = 8 for males and *n* = 8 for females for PS19_shBSN_ mice (**c**-**f**), and *n* = 4 per sex (**g**-**j**). Significance was determined by one-way ANOVA (**a**–**f**), and unpaired two-tailed Student’s t-test (**g**–**j**). Source data

A second cohort of 6-month-old PS19 and WT littermates were injected at P0 with scramble or *Bsn* shRNA to determine if this synaptic integrity rescue represented a functional improvement. We measured long-term potentiation (LTP) in the CA1 hippocampal area in mouse brain slices. We monitored field excitatory postsynaptic potentials (fEPSPs) evoked by extracellular stimulation of the Schaffer collateral pathway and induced LTP using high-frequency stimulation (four stimuli of 100 Hz for 1 s with a 10-s interstimulus interval). Severely impaired hippocampal LTP was observed in PS19_scramble_ mice versus WT_scramble_ and WT_shBSN_ mice; however, a decrease in BSN levels in PS19_shBSN_ mice rescued LTP (Fig. 6a–c). We observed no difference in LTP between the PS19_shBSN_ mice and the WT_scramble_ or WT_shBSN_ controls. We also measured the paired-pulse ratio (PPR), which decreased in PS19_scramble_ mice versus WT_scramble_ and WT_shBSN_ animals, reflecting diminished synaptic vesicular release probability. This reduction was rescued in PS19_shBSN_ animals (Fig. 6d). The beneficial effect of *Bsn* downregulation on LTP and PPR was observed in both female and male PS19 mice (Extended Data Fig. 9a,b).

**Fig. 7:**
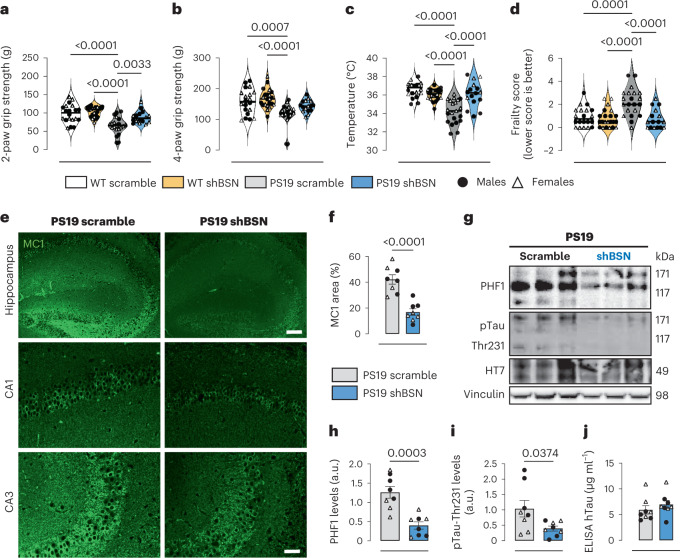
Reducing bassoon levels improves behavioral deficit and diminishes pathological tau species in a late stage of pathology. **a**–**d**, Two-paw test (**a**), four-paw test (**b**), body temperature (**c**) and frailty test (**d**) in 9-month-old WT and PS19 mice injected with shBSN or scramble shRNA. **e**, Hippocampal MC1 immunostaining in 9-month-old PS19 mice injected with shBSN and scramble shRNA. Scale bars, 200 µm and 50 µm for CA1 and CA3 insets, respectively. **f**, Quantification of MC1 immunostaining in shBSN and scramble PS19 mice as a percentage of area. **g**–**i**, Western blot (**g**), and quantification of specific antibodies against pTau-Ser396/Ser404 (PHF1; **h**), and pTau-Thr231 (**i**), in shBSN and scramble PS19 mouse brain lysates. **j**, Total human tau levels by ELISA in shBSN and scrambled PS19 mouse brain lysates. Experiments were performed with *n* = 20 for WT_scramble_, *n* = 21 for PS19_scramble_, *n* = 22 for WT_shBSN_ and *n* = 16 for PS19_shBSN_ mice (**a**–**d**), and *n* = 8 (**e**–**j**). Significance was determined by one-way ANOVA (**a**–**d**) and unpaired two-tailed Student’s *t*-test (**f** and **h**–**j**). Bar graphs with error bars indicate the mean ± s.e.m. Source data

A third cohort of 9-month-old mice was subjected to behavioral and physiological tests to further assess the functional benefit of *Bsn* downregulation. As we recently demonstrated a decrease in motor strength in PS19 mice^39^, the grip strength of the front two paws and all four paws were tested. PS19_scramble_ mice exhibited reduced two-paw and four-paw grip strength compared to WT_scramble_ and WT_shBSN_ mice (Fig. 7a,b). There were no differences between PS19_shBSN_ mice and WT_scramble_ or WT_shBSN_ mice, suggesting that *Bsn* downregulation rescues motor impairment. The physiological characteristics were evaluated by measuring the basal core body temperature and frailty markers. PS19_scramble_ mice had lower basal body temperatures than the WT_scramble_ and WT_shBSN_ mice; however, downregulation of Bsn levels completely rescued the decreased body temperature in PS19_shBSN_ mice (Fig. 7c). We then performed a clinical exam to assess the 26 frailty parameters^39^. PS19_scramble_ mice exhibited more frailty markers than WT_scramble_ and WT_shBSN_ mice (Fig. 7d), indicating a decline in general health. No differences were observed between PS19_shBsn_ mice and either WT group, indicating the benefit of *Bsn* downregulation in the context of tau pathology. Motor and physiological rescue by *Bsn* downregulation were observed in both male and female PS19 mice (Extended Data Fig. 9c–f).

**Extended Data Fig. 10:**
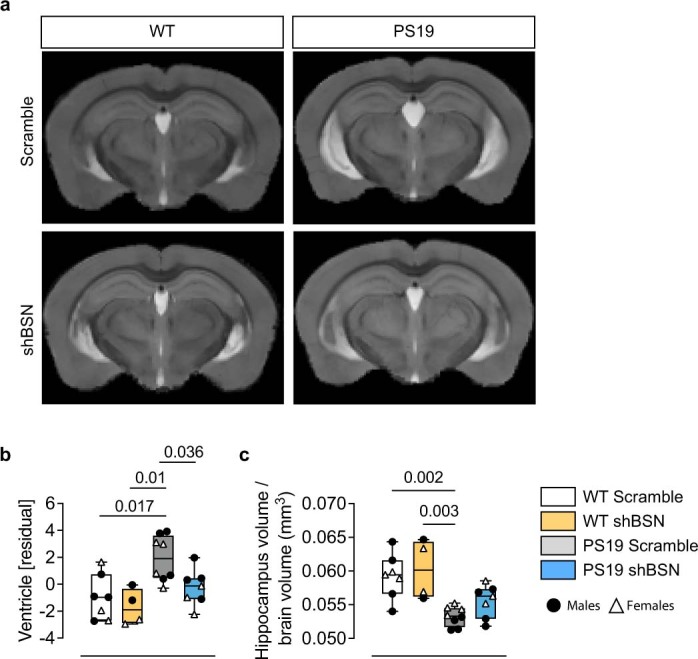
Downregulation of bassoon corresponds to reduced ventricular volume and an increment of hippocampal volume in PS19 mice. **a**–**c**, *In vivo* Magnetic resonance imaging (MRI) (**a**), quantification of ventricular (**b**), and hippocampal volume normalized to total brain volume (**c**), in 9-month-old WT and PS19 mice injected with shBSN or scramble shRNA. Data represent the mean ± s.e.m. Experiments were performed with *n* = 7 for WT_scramble_, *n* = 8 for PS19_scramble_, *n* = 4 for WT_shBSN_, and *n* = 7 for PS19_shBSN_ mice. Significance was determined by one-way ANOVA **(b, c)**. **b**, **c**, for box plots, midlines represent the median, boundaries are interquartile range, and the whiskers are minimum-maximum. Source data

We next tested whether *Bsn* downregulation could reduce neurodegeneration in PS19 mice. As rising ventricular volumes and a decline in hippocampal volume indicate brain atrophy in 9-month-old PS19 mice^40^, we determined the effect of *Bsn* downregulation on ventricular and hippocampal volume by volumetric magnetic resonance imaging MRI analysis. *Bsn* downregulation did not significantly alter the ventricular nor hippocampal volume in WT mice (Extended Data Fig. 10a–c). PS19_scramble_ mice exhibited an increase in ventricular volume and a decline in hippocampal volume compared to the WT_scramble_ and WT_shBSN_ mice (Extended Data Fig. 10a–c). However, decreasing BSN levels in PS19 mice significantly ameliorated the ventricular volume increase compared to PS19_scramble_ mice (Extended Data Fig. 10a–c). No differences were observed in hippocampal volume between PS19_shBSN_ mice and WT_scramble_ or WT_shBSN_ mice, suggesting that *Bsn* downregulation partially reduces neurodegeneration in tauopathies. Due to the limited number of mice, it was not possible to determine sex differences in the beneficial effects of *Bsn* downregulation on restoring ventricular and hippocampal volume. Finally, we evaluated whether the functional improvement and rescue of brain atrophy observed in 9-month-old PS19 mice were associated with decreased pathological tau burden. Immunofluorescence analysis revealed that *Bsn* downregulation reduced tau pathology in the hippocampus of PS19 mice (Fig. 7e,f). Using western blotting, we also confirmed that downregulation of *Bsn* decreased phosphorylated tau aggregates (Fig. 7g–i) but did not affect total tau levels measured by ELISA in brain samples from 9-month-old PS19 mice (Fig. 7j). The decrease in pathological tau burden was significantly observed in 9-month-old female and male PS19 mice (Extended Data Fig. 9g–j), suggesting that the effect of *Bsn* downregulation on early stages of tau aggregation (Fig. 5) has lasting effects on tau burden when higher levels of pathology have developed.

## Discussion

We identified BSN as a tau-seed interactor exhibiting a preponderant role in tau-seed stability and spreading. Downregulating *Bsn* significantly reduced tau spreading and overall tau pathology, improved synaptic integrity, rescued electrophysiological and behavioral impairments and reduced brain atrophy. BSN is a large scaffolding protein (419 kDa) of the presynaptic active zone involved in the regulation of neurotransmitter release at the synapse^22^. Mice with constitutive ablation of the bassoon gene show no abnormalities in brain architecture but have impaired presynaptic functions^41^. Interestingly, partial loss of *Bsn (Bsn*^+/−^*)* causes no abnormalities, suggesting that a 50% decrease in *Bsn* levels is well tolerated^41^. The recently developed *Bsn*^*floxP/floxP*^ mouse model^34^ shows no neuronal differences from constitutive knockout allele by Cre-mediated recombination in the germ line and WT controls, suggesting that ablation of *Bsn* is not detrimental. BSN also regulates presynaptic ubiquitination, proteostasis and autophagy^29,30^. Synaptic accumulation of tau aggregates in AD is associated with dysfunction of the ubiquitin–proteasome system, suggesting that these aggregates may be an important mediator of the proteotoxicity that disrupts synapses in AD^42^. BSN is also involved in regulating neurotransmitter release from glutamatergic synapses^41^. The selective ablation of *Bsn* in excitatory neurons enhances learning performance in mice^22^. Little is known about the importance of BSN in tau pathology and neurodegeneration. The strongest evidence linking BSN with tau pathology was published in 2018, revealing four missense mutations in the BSN gene (p.Pro3866Ala) and aggregation of three-repeat and four-repeat tau in patients^32^. Another study revealed increased BSN expression in patients with multiple system atrophy^33^, a neurodegenerative disease characterized by the aggregation of alpha-synuclein and tau protein^43^. A new study recently demonstrated the toxic accumulation of BSN in the neuronal somata of mice and patients with multiple sclerosis^34^. Notably, this study also demonstrated that the genetic ablation of *Bsn* protected mice from inflammation-induced neuroaxonal injury and enhanced neuronal survival^34^. Overall, these studies suggest associations among BSN, neurodegenerative events and tau pathology, and support the feasibility of BSN downregulation as a therapeutic avenue for neurodegenerative tauopathies.

Apart from BSN, we also identified other presynaptic interactors. The association between the tau seed and presynaptic proteins is relevant considering that numerous studies have suggested that secreted tau can spread among synaptically connected neurons^5,44^. Tracy et al.^21^ recently demonstrated that WT tau interacts with numerous presynaptic proteins at the active zone in human induced pluripotent stem cell-derived neurons. The authors demonstrated that neuronal activity enhanced the interaction of tau with synaptic vesicle proteins, suggesting that presynaptic fusion machinery could regulate activity-dependent tau release through direct protein–protein interaction. The authors observed that tau interacts with synaptotagmin-1 (SYT1) and an increase in neuronal activity enhanced the interaction of tau with SYT1. Interestingly, we identified SYT1 as an interactor of the tau seed (Supplementary Data 2), supporting the notion that the tau seed interacts with presynaptic proteins strongly associated with physiological tau during neuronal activity. In our study, we also identified synaptogyrin-3 (SYNGR3) as an interactor of the tau seed and monomeric tau (Supplementary Data 2). Additionally, it has been previously shown that tau interaction with SYNGR3 inhibits presynaptic vesicle release, and SYNGR3 downregulation rescues tau-induced defects in a mouse model of tauopathy^11^, supporting the beneficial effects of downregulating the levels of a presynaptic tau interactor. Together with previous work, our study supports the relevance of presynaptic tau interactors in disease pathogenesis via a diverse set of mechanisms that are not mutually exclusive. One mechanism could involve increased stability or resistance to degradation of a toxic tau conformer due to its interaction with presynaptic proteins. Another possibility involves the loss of physiological synaptic functions due to the interaction of these synaptic proteins with tau aggregates. A third possibility is that a tau conformer with seeding activity utilizes the presynaptic vesicle fusion machinery for neuronal release, as suggested for physiological tau^21^, through aberrant protein interactions. In our study, we focused on the presynaptic protein BSN as a new interactor of the tau seed, considering the strong genetic link between BSN mutation and human tauopathies^32^. Nevertheless, further studies are necessary to determine the relevance of the presynaptic machinery in tau-seed propagation and overall pathogenesis.

The formation of pathological aggregates is likely driven by multiple processes involving complex interactions between the disease-associated proteins and other proteins^45^. It has been proposed that flexible hydrophobic surfaces enable soluble aggregates to engage in aberrant interactions with metastable proteins that share distinct physicochemical properties^37^. Proteins prone to interact with aggregates are usually larger, have a lower average hydrophobicity and exhibit high structural flexibility; additionally, they have significant enrichments in intrinsically disordered regions^37^. BSN, a large multidomain protein with long stretches of intrinsically disordered residues, is particularly prone to accumulation^34^; thus, BSN could interact with amyloid-like aggregates. Previous studies identified tau interactors from total cellular or brain lysate without discerning between tau aggregation state^15–21^, which may have masked the effect of proteins, such as BSN, that solely interact with the tau seed. Amyloid-like aggregates were originally reported to exert toxicity, in part, by interacting with and sequestering functional proteins, leading to the collapse of essential cellular functions^37^. Nevertheless, it was recently suggested that tau aggregates in the brain are stabilized by unknown cofactors that lead to higher-quality packing^46^. Our results indicate that BSN might contribute to tau pathogenesis by promoting the stabilization and resistance to degradation of abnormal tau structures and assemblies with strong seeding activity. Nonetheless, we cannot rule out that BSN dysfunction affects the regulation of neurotransmitter release^41^ or presynaptic proteostasis^29,30^, which could contribute to tau-related toxicity.

Recent breakthroughs in cryo-EM have yielded atomic structures of tau filaments upon sarkosyl extraction from brains of individuals with various tauopathies, demonstrating that each disease is characterized by a unique tau filament fold^47^. Considering this structural heterogeneity between tauopathies, further studies using approaches such as cryo-EM and immuno-EM^48^ would be important to determine structural and morphological differences between tau seeds isolated from patients with AD, patients with PSP and PS19 mice.

In summary, our findings improve the understanding of the nature of the tau seed, highlighting the importance of identifying interactors, such as BSN, that could work as scaffolds or stabilizers of the pathogenic seed. The inhibition of these interactions could be a new therapeutic approach for neurodegenerative tauopathies.

## Methods

### Mice

All work involving mice was performed in accordance with the Guide for the Care and Use of Laboratory Animals (National Institutes of Health (NIH)) and procedures approved by the Indiana University School of Medicine (IUSM) Institutional Animal Care and Use Committee. Mice were bred and housed at the IUSM animal care facility and were maintained according to US Department of Agriculture standards (12-h light/dark cycle, food and water ad libitum, ~25 °C, 40–60% humidity) in accordance with the Guide for the Care and Use of Laboratory Animals (NIH). The PS19 mouse model, which overexpresses human 1N4R tau with the P301S mutation on the C57B6/J background, was directly purchased from The Jackson Laboratory (stock no. 008169). For all described experiments, 4-month-old, 6-month-old and 9-month-old PS19 and WT littermates of both sexes were utilized. All mice were randomly assigned to treatment and experimental groups.

### Human tissue samples

Frozen samples consisting of blocks of postmortem brain tissues from individuals with AD, individuals with PSP and controls were provided by the Brain Resource Center at Johns Hopkins. AD cases consisted of pathologically severe AD, stage V–VI (Supplementary Data 1).

### Preparation of Tris-buffered saline-soluble homogenates

Each brain tissue was homogenized in TBS buffer at a ratio of 1:10 (wt/vol) with a protease inhibitor cocktail (Roche, 11873580001). Samples were then centrifuged at maximum speed for 15 min at 4 °C. The supernatants were portioned into aliquots, snap frozen and stored at −80 °C until analyzed.

### Size exclusion chromatography

SEC was performed with a Superose 6 Increase 10/300 GL column (GE Healthcare, 29091596) on an ÄKTA pure 25 L chromatography system (GE Healthcare, 29018224). The column was equilibrated with 1.5 CV of a 50 mM NaCl, 50 mM Tris pH 8.0 buffer at a flow rate of 0.7 ml min^−1^. Samples were clarified by centrifugation at 10,000*g* for 10 min. Protein concentration was quantified by Bradford assay, and 1–5 mg total protein of supernatant was taken for separation, depending on the sample. The supernatant was concentrated with a 0.5-ml 3K Amicon centrifugal filter (Millipore Sigma, UFC5003) to ~200 µl, then loaded onto the column via sample loop injection. Starting from injection, 1-ml fractions were collected into tubes containing EDTA-free protease inhibitor (Roche, 11873580001) at a flow rate of 0.3 ml min^−1^.

### Tau-seeding assay

The seeding assay was performed as previously described^24^ with minor modifications. TauRD P301S FRET Biosensor cells (American Type Culture Collection (ATCC), CRL-3275) were plated at 35,000 cells per well in 130 µl medium in a 96-well plate, then incubated at 37 °C overnight. The next day, cells were transfected with cell or brain lysate (20 µg total protein per well) by using Lipofectamine 2000 and then incubated at 37 °C for 48 h. Cells were harvested by trypsinization. Flow cytometry was conducted with a BD LSRFortessa X-20 with a High Throughput Sampler, using the BD FACS Diva (v8.0) software. FlowJo (v10.0) was used for data analysis. The BV421 channel (excitation of 405 nm, emission of 450/50 nm) was used to detect CFP, and the BV510 channel (excitation of 405 nm, emission of 525/50 nm + 505LP) was used to detect FRET signal, with compensation to remove the CFP spillover into the FRET channel. Data analysis was performed with FlowJo, using the gating strategy shown in Extended Data Fig. 1a. Seeding was quantified by integrated FRET density, defined as the product of the percentage of FRET-positive cells and median fluorescence intensity of FRET-positive cells.

### Human tau ELISA

ELISA was performed on SEC fractions using Tau (Total) Human ELISA Kit (Invitrogen, KHB0041) by following the directions provided by the manufacturer. Lysates were diluted 1:50,000 in blocking buffer. F7–F14 were diluted at a ratio of 1:2,000 in blocking buffer. F15–F22 were diluted at a ratio of 1:20,000 in blocking buffer.

### Guanidine hydrochloride denaturation assay

Each sample was diluted to the same protein concentration by Bradford assay. Samples were denatured by adding a Gdn-HCl solution at a 1:1 ratio by volume, varying the initial concentration of the Gdn-HCl solution to obtain the desired final concentration after mixing. The mixture was incubated for 30 min at room temperature (RT). After incubation, the mixture was diluted for ELISA or western blot immediately. To control for the potential effect of trace amounts (<5 mM) of Gdn-HCl on the ELISA assay, a 1 ng ml^−1^ tau standard was also spiked with Gdn-HCl at a concentration matching that of each sample. The effect of trace Gdn-HCl on the assayed ELISA concentration was not statistically significant.

### Immunoprecipitation

From HEK293T lysates, SEC fraction 9 from PS19 mice, and AD and PSP human samples, tau was immunoprecipitated by using 2 µg of biotinylated HT7 antibody (Thermo Fisher, MN1000b), for every 100 ng of human tau quantified by ELISA. IgG isotype control antibodies (BioLegend, 400104) were used for comparison. The IP and flow-through samples were then subjected to further analysis.

### Electron microscopy

IP samples were analyzed by negative-stain biological transmission EM as described before^49^. Briefly, 3 µl of the IP sample was directly pipetted on discharged carbon-coated copper transmission EM grids and incubated for 1 min. Grids were carefully washed with deionized water without letting it dry and stained with 3.5 μl of 1% (wt/vol) phosphotungstic acid solution for 1 min. Any excess solution was then removed by blotting with a Whatman filter paper. The samples were imaged using an FEI Tecnai T12 transmission electron microscope operating at 80 kV. Images were captured using Gatan digital micrograph software and the width was measured using image analysis software ImageJ. Three independent samples were analyzed, and results were plotted with Prism 9.0 software.

### Mass spectrometry

Sample preparation for the affinity purification mass spectrometry experiments was designed at the Proteomics Core Facility of IUSM from previously reported qPLEX-RIME methodology that have integrated isobaric labeling and tribrid mass spectrometry methods with RIME (Rapid Immunoprecipitation Mass spectrometry of Endogenous proteins) with modifications^50–52^. Briefly, magnetic beads, affinity captured with proteins, were treated with 10 μl trypsin/LysC (15 ng µl^−1^; Promega Corporation) in 100 mM ammonium bicarbonate and incubated overnight at 37 °C with shaking, followed by a second digestion with trypsin/LysC at RT for 4 h. The magnetic beads were next separated on a magnetic stand to separate the supernatant peptide solution. De-salting was carried out using Sep-Pak Vac 1 cc C18 cartridges (Waters Corporation) using a vacuum manifold. All samples were then dried using a SpeedVac, reconstituted in 50 mM triethylammonium bicarbonate and subjected to tandem mass tags (TMT)-based labeling using TMT10plex reagents (Thermo Fisher, 90309). Next, Nano-LC–MS/MS analyses were performed on an Orbitrap Fusion Lumos mass spectrometer (Thermo Fisher Scientific) coupled to an EASY-nLC HPLC system (Thermo Scientific). Labeled, mixed and dried peptide samples were reconstituted in 0.1% formic acid (FA; 20 μl) and 8 μl equivalent volume was loaded onto a reversed-phase PepMap RSLC C18 column (2 μm, 100 Å, 75 μm × 50 cm) with Easy-Spray tip using an applied maximum pressure of 750 bar. The peptides were eluted using a varying mobile phase (MP) gradient from 94% phase A (FA/H_2_O 0.1/99.9, vol/vol) to 28% phase B (FA/acetonitrile 0.1/99.9, vol/vol) for 160 min; to 35% phase B for 5 min; to 50% phase B for 14 min to ensure elution of all peptides and bringing down the MP composition to 10% phase B for 1 min at 400 nl min^−1^ to bring the MP composition to a higher percentage of phase A. Nano-LC MP was introduced into the mass spectrometer using an EASY-Spray Source (Thermo Scientific). During peptide elution, the heated capillary temperature was kept at 275 °C and ion spray voltage was kept at 2.6 kV. The mass spectrometer method was operated in positive ion mode for 180 min with a cycle time of 4 s. Mass spectrometry data were acquired using a data-dependent acquisition method that was programmed to have two data-dependent scan events following the first survey mass spectrometry scan. During MSn level 1, using a wide quadrupole isolation, survey scans were obtained with an Orbitrap in the range of 375–1,500 *m/z* at 60,000 resolution. To isolate and fragment the selected precursor ions, MSn level 2 scans were performed with following vendor defined parameters: isolation mode, quadrupole; isolation offset, off; isolation window, 0.8; multi-notch isolation, false; scan range mode, auto normal; first mass, 100; activation type, higher-energy collisional dissociation; collision energy mode, fixed; collision energy (%), 38; detector type, orbitrap; orbitrap resolution, 50,000; maximum injection time, 90 ms; automatic gain control target, 1E5; data type, centroid; polarity, positive; source fragmentation, false. The data were recorded using Thermo Scientific Xcalibur (4.1.31.9; 2017) software. Resulting RAW files were analyzed using Proteome Discover 2.2.0.388 (Thermo Fisher Scientific). The MS/MS spectra were searched against in silico tryptic digest of a database (FASTA) downloaded from UniProt (mouse_human_uniprot_contam_030419.fasta) using the SEQUEST HT search engine. The following search parameters were applied: trypsin as the proteolytic enzyme; peptides with a maximum number of 2 missed cleavages, precursor mass tolerance of 10 ppm, and a fragment mass tolerance of 0.6 Da. Static modifications used for the search were: (1) carbamidomethylation on cysteine residues; (2) TMT sixplex label on lysine residues and the N termini of peptides. Dynamic modifications used for the search were: oxidation of methionines and phosphorylation of S/T/Y. Percolator false discovery rate (pFDR) was set to a strict setting of 0.01 and a relaxed setting of 0.05. Values from both unique and razor peptides were used for quantification.

#### Enrichment analysis

Abundance ratios of HT7 IP were compared with control IgG IP to determine protein enrichment levels (*n* = 3). Proteins enriched by a fold change > 4 with *P* < 0.05 by *t*-test (total of 1,159 in tau seed and 365 in tau monomer) were selected for DAVID functional annotation (https://david.ncifcrf.gov/) using default settings with the mouse genome. We then compared both interactomes with the ‘Wall of Targets’ nominated by the AMP-AD as possible targets for AD (https://agora.ampadportal.org/genes/(genes-router:genes-list)). Synaptic proteins were identified by searching Gene Ontology for genes that have a ‘part of’ or ‘regulate’ transitive closure relation with the synapse term (GO:0045202).

### Immunohistochemistry and proteinase K sensitivity assay

Slides were deparaffinized and rehydrated in several incubations of xylene, ethanol gradient (100% to 30%) and deionized water. The slides were stained with hematoxylin and eosin (Vector Labs, H-3502) following the manufacturer’s protocol. Slides were subsequently dehydrated, coverslipped and imaged.

For PK sensitivity assay, following antigen retrieval, the slides were incubated with 50 μg ml^−1^ PK (Bioline, BIO-37037), 10 mM Tris HCl pH 7.8, 100 mM NaCl, 0.1% NP-40 at 37 °C for 0 s, 10 s and 45 s, similarly to what was described before^53^.

### Disorder and hydrophobicity profile

Disordered residues were predicted by ESpritz^54^ using the DisProt database. The optimal binary decision threshold *S*_*w*_ was used as the threshold for disorder. The hydrophobicity profile was calculated using the method of Kyte & Doolittle^55^ with a moving average window of nine residues.

### Immunofluorescences

Mouse paraffin sections were deparaffinized in xylene, rehydrated in ethanol gradient (100% to 30%) and washed with deionized water. Then, the sections were heated to 95 °C in high pH antigen retrieval solution (Invitrogen eBioscience, 00-4956-58) for 10 min with a microwave oven. After washing twice with PBS (5 min each), the sections were incubated with TrueBlack (Biotium, 23007) for 3 min and then washed three times in PBS. Then, tissues were blocked with PBS 10% goat serum and 0.01% Triton X-100 for 1 h at RT. Sections were then incubated overnight at 4 °C with the following primary antibodies: HT7 (Thermo Fisher, MN1000; 1:300 dilution), anti-Bassoon (Millipore, ABN255; 1:300 dilution), anti-GFP (Abcam, ab1218; 1:1,000 dilution), MC1 (Peter Davies; 1:300 dilution), PHF1 (Peter Davies; 1:300 dilution), anti-GFAP (Sigma-Aldrich, G3893; 1:100 dilution), anti-IBA1 (Wako, 019-19741; 1:100 dilution)), 6x-His (Thermo Fisher MA1-1351:300), PSD95 (Abcam, ab2723; 1:100 dilution) and Syn-1 (Abcam, ab64581; 1:100 dilution). The next day, sections were quickly washed three times in PBS and incubated for 2.5 h with a 1:500 ratio of Alexa Fluor antibodies (goat anti-rabbit Alexa Fluor 488 (Invitrogen, A11008; 1:200 dilution), goat anti-mouse Alexa Fluor 488 (Invitrogen, A32723; 1:200 dilution), goat anti-rabbit Alexa Fluor 568 (Invitrogen, A11036; 1:200 dilution), goat anti-mouse Alexa Fluor 568 (Invitrogen, A11031,1:200 dilution)), diluted in blocking solution. Sections were quickly washed three more times in PBS and mounted with Fluoromount (Sigma, F4680). For AD and PSP cases, frozen sections were first fixed with 4% paraformaldehyde for 1 h at RT and then permeabilized in Triton X-100 0.01% for 1 h, continuing with the mentioned immunofluorescence protocol. For cell immunofluorescences, HEK293T cells were fixed in 4% paraformaldehyde for 30 min and washed three times for 5 min with 1× PBS. Cells were permeabilized with 0.01% Triton X for 20 min at RT and washed three times with 1× PBS. Coverslips were incubated with 10% Normal Goat Serum and blocked for 30 min at RT. Then, the coverslips were incubated with primary antibodies (MC1 (Peter Davies; 1:300 dilution) and anti-Bassoon (Millipore, ABN255; 1:300 dilution)) diluted in 10% normal goat serum overnight at 4 °C. The next day, coverslips were washed three times with 1× PBS and incubated with Alexa secondary antibodies (goat anti-mouse Alexa Fluor 488 (Invitrogen, A32723; 1:200 dilution) and goat anti-rabbit Alexa Fluor 568 (Invitrogen, A11036; 1:200 dilution)), diluted in 10% normal goat serum for 2 h at RT. Coverslips were then washed three times with 1× PBS and mounted using Fluoromount (Sigma, F4680).

### Proximity ligation assay

HEK293T cells (ATCC, CRL-3216) were grown on coverslips and transfected with plasmids encoding Tau^WT^, Tau^P301S^, full-length BSN^WT^ and BSN N-terminal and C-terminal fragments. At 72 h later, cells were fixed in 4% paraformaldehyde for 30 min and washed three times for 5 min with PBS. PLA was performed using the Duolink In Situ Fluorescence kit (Sigma, DUO92101) according to the manufacturer’s instructions. Briefly, the coverslips were incubated with the Duolink Blocking solution for 1 h at 37 °C in a humidified chamber. Then, the blocking solution was removed, and coverslips were incubated with primary antibodies (MC1 (Peter Davies; 1:300 dilution), anti-Bassoon (Millipore, ABN255; 1:300 dilution), anti-total tau (DAKO, A0024; 1:300 dilution) and 6x-His (Thermo Fisher, MA1-135; 1:300 dilution)) diluted in Duolink Antibody Diluent overnight at 4 °C. The coverslips were then washed with wash buffer A (Sigma) twice for 5 min. Then, the Duolink In Situ PLA Probe Anti-Rabbit PLUS and Duolink In Situ PLA Probe Anti-Mouse MINUS PLA probes (Sigma) diluted in Duolink Antibody Diluent were applied to the coverslips and incubated in a humidified chamber for 1 h at 37 °C. Coverslips were washed twice with wash buffer A, followed by incubation with the ligation solution for 30 min at 37 °C, then washed twice with buffer A and incubated with amplification solution for 100 min at 37 °C. Finally, the coverslips were washed twice for 10 min in buffer B (Sigma), once in 0.01% buffer B and then mounted onto the slides using the Duolink In Situ Mounting Medium with DAPI (Sigma). The edges were sealed with clear nail polish.

### Imaging and analysis

Mice sections were imaged with a Nikon A1-R laser scanning confocal microscope coupled with Nikon AR software. For tau propagation analysis, images (*z*-stack step size of 0.5 µm) were imported into ImageJ (NIH). Propagation analysis was performed by counting HT7^+^/GFP^−^ or GFP^+^ cells per 1 mm^2^. For functional synapsis analysis, image stacks (0.1 µm) were imported into ImageJ, and colocalization was quantified by Pearson’s correlation coefficient using the JACoP plug-in.

### Cell culture co-transfection

HEK293T cells (ATCC, CRL-3216) were cultured in DMEM (Invitrogen) with 10% FBS (Invitrogen). Human Tau-P301L/WT and WT human Bassoon were cloned in pRK5 and CMV plasmid (Vector Builder), respectively. Plasmids were transfected, in 1:10 dilution of Tau:BSN, with Lipofectamine 3000 (Invitrogen) and incubated for 72 h. Cells were lysed in 1× TBS with protease inhibitor (Roche) by sonication (2 min, 30% Amp, 5 s ON and 5 s OFF). The lysate was centrifuged at 21,100*g* for 10 min at 4 °C. The supernatant (TBS-soluble fraction) was transferred into fresh tubes and used for downstream seeding activity, western blot and IP analysis.

### Western blot analysis

TBS-soluble samples were incubated with reducing 6× Laemmli SDS buffer (Alfa Aesar, J61337) at 95 °C for 10 min for denaturing conditions or 4× NuPAGE sample buffer (Invitrogen NP0007) for non-denaturing conditions and then loaded on 4–12% NuPAGE Novex gels (Invitrogen). Nitrocellulose membranes were used to transfer proteins and blocked with 5% BSA in TBS with 0.01% tween followed by overnight incubation of primary antibodies (HT7 (Thermo Fisher, MN1000; 1:1,000 dilution), anti-Bassoon (Millipore, ABN255; 1:1,000 dilution), MC1 (Peter Davies; 1:1,000 dilution), anti-total tau (DAKO, A0024; 1:5,000 dilution), PHF1 (Peter Davies; 1:1,000 dilution), anti-Actin (Abcam, ab8227; 1:2,000 dilution), pThr231 (Millipore, MAB5450; 1:1,000 dilution) and Vinculin (Sigma, V9131; 1:1,000 dilution)) diluted in the blocking solution. Horseradish peroxidase (HRP) secondary antibodies (goat anti-mouse HRP conjugated (Invitrogen, 626820; 1:5,000 dilution) and goat anti-rabbit HRP conjugated (Invitrogen, 31460; 1:5,000 dilution)) were incubated for 1 h at RT and the proteins were detected with Supersignal West Pico (Thermo Scientific, 34580) and imaged by using iBright 1500 (Invitrogen). Western blots were analyzed using ImageJ (NIH, v1.53i).

### *Drosophila* stocks and genetics

*D. melanogaster* stocks and crosses were maintained on Nutri-fly Bloomington formulation (Genesee Scientific) at 25 °C in a 12-h light/dark cycle. All the fly experiments were performed at day 30 after eclosion. Transgene overexpression was achieved with the *Gal4/UAS* system, using GMR-Gal4 and *GMR-Tau*^*P301L*^ lines (Bloomington Drosophila Stock Center, 9146 and 51377, respectively)^56^. For human BSN overexpression, full-length BSN WT and p.Pro3866Ala mutant sequences were cloned downstream of the Gal4-responsive upstream activating sequences into the pUAST plasmid (VectorBuilder) and then microinjected in fly embryos (BestGene).

#### Eye phenotype quantification, western blot and seeding

For light microscope imaging, adult flies were immobilized at −80 °C, and then mounted for visualization. Flies were then imaged using a Leica DMC6200 camera with a ×10 objective, with a white light falling on each ommatidium until observing a single reflection spot in its center. Images were captured and stacked using Zerene Stacker (v1.01, Zerene Systems). For eye phenotypic scoring, the Flynotyper plug-in was used on ImageJ as previously described^57^.

### Adeno-associated virus production and injections

For shRNA, sequences for mouse *Bsn* shRNA (CCTAACGCTTTCCTCTGACAT) and scramble shRNA (CCTAAGGTTAAGTCGCCCTCG) were used. shRNA sequences were cloned downstream of the U6 promoter and packaged into AAV9 from VectorBuilder.

For in vivo tau propagation experiments, the EGFP-P2A-hTau(2N4R)^P301L^ sequence was cloned downstream of the CMV promoter and packaged into AAV9 (VectorBuilder). For *Bsn* downregulation, neonatal (P0) PS19 and WT mice were injected with the AAV scramble or shRNA *Bsn* sequence. Animals were euthanized at 4, 6 or 9 months after injections. For in vivo tau propagation experiments, neonatal WT C57B6/J mice were injected with AAV encoding either scramble or shRNA *Bsn* sequences (*n* = 4 for each group, 2 males and 2 females per group). After 12 weeks, mice were intracranially injected with an AAV encoding EGFP-P2A-hTau(2N4R)^P301L^ into the hippocampus (1 μl in the left hemisphere). Mice were anesthetized with isoflurane (2%) and AAV injections at a rate of 0.5 μl min^−1^ were made at the following coordinates from bregma: A/P, −2 mm; M/L, −1.5 mm; D/V, −1.5 mm. Head skin was sutured, and mice were allowed to recover on a warming incubator. Mice received meloxicam for 2 d after surgery.

### Electrophysiology

Six-month-old WT (10–11 males and 10–11 females) and PS19 (10–13 males and 9–11 females) littermates were used. Hippocampal slices of 300 µm in thickness were cut at 0.1 mm s^−1^ with a Leica VT1200 vibratome in ice-cold oxygenated external solution containing a sucrose-based artificial cerebrospinal fluid (aCSF; 194 mM sucrose, 30 mM NaCl, 26 mM NaHCO_3_, 10 mM glucose, 4.5 mM KCl, 0.5 mM NaH_2_PO_4_ and 1 mM MgCl_2_, pH 7.4) bubbled with 95% O_2_/5% CO_2_. Before cutting, mice were anesthetized with isoflurane and perfused transcardially with 10 ml of cold aCSF solution. The brains were quickly removed and blocked. After cutting, slices were transferred to an incubation chamber containing oxygenated aCSF solution at 33 °C for 1 h. Slices were then kept at RT until recording. Recordings were done at 30–32 °C in a submersion chamber perfused (1–2 ml min^−1^) with aCSF solution comprising 124 mM NaCl, 4.5 mM KCl, 1.2 mM NaH_2_PO_4_, 1 mM MgCl_2_, 2 mM CaCl_2_, 26 mM NaHCO_3_ and 10 mM glucose, continuously bubbled with 95% O_2_/5% CO_2_ at a pH of 7.4 and 310 mOsm.

#### Input–output, paired-pulse ratio and long-term potentiation recordings

For input–output curves and LTP experiments, fEPSPs evoked by Schaffer collateral stimulation with stainless-steel stereotrodes (1 MΩ, P1 Technologies) were recorded in current-clamp mode with micropipettes filled with 1 M NaCl using a Multiclamp 700B amplifier and Clampex software (Molecular Devices). Signals were low-pass filtered at 2 kHz and digitized at 50 kHz. The recording micropipette was placed in the CA1 region of the hippocampus. The intensity of the stimulator was increased stepwise until a maximal response was obtained using a constant current isolated stimulator (Digitimer). The slope of the fEPSP (mV/ms) was measured. PPR measurements were obtained every 20 s at a 40-ms interstimulus interval. The LTP protocol consisted of 10 min of stable baseline: 30 pulses every 20 s (stimulus strengths were <50% of the strength evoking a maximal response); 1 min conditioning: trains (10 pulses at 100 Hz) repeated four times every 20 s; then a 60 min post-conditioning period at the same baseline stimulation frequency: The synaptic strength change was measured from the slope of the fEPSP and data were expressed as a percentage of change with respect to the average baseline.

### Behavioral test

Nine-month-old PS19 (17 males and 20 females) and WT (23 males and 19 females) littermates were used. Mice were weighed, and the resting core body temperature was measured by inserting a lubricated rectal probe (Bioseb) ~1 cm into the rectum for 10 s. For the frailty examination, mice were assessed for the presence or absence of 26 different characteristics as described before^58^, with modifications. A score of 0 was given if the mouse had no sign of the deficit, 0.5 if there was a mild deficit and 1 if there was a severe deficit. Grip strength of the forelimbs (front two paws) and all limbs (four paws) was evaluated using the Grip Strength Meter (Bioseb, BIO-GS3). Following the manufacturer’s protocol, mice were held by the tail and lowered toward the apparatus and allowed to grab the metal grid using two or four paws. The mice were pulled backward horizontally, and the force applied to the grid just before they lost their grip was recorded as the peak tension (converted to grams by the transducer). Peak force was measured twice in succession for each mouse for the front two paws and all four paws. The mean value of both trials was used for analysis. Mice were given a minimum break of 5 min between trials.

### Magnetic resonance imaging

Nine-month-old PS19 (eight males and seven females) and WT (six males and five females) littermates were used for MRI. T2-weighted high-resolution structural images were acquired in a horizontal bore 9.4 Tesla BioSpec preclinical MRI system (Bruker BioSpin MRI, Germany) equipped with shielded gradients (maximum gradient strength, 660 mT/m; rise time, 4,570 T/m/s) and 1H mouse cryogenic surface coil (Cryoprobe, Bruker, Biospin). Two-dimensional T2-weighted (Bruker, T2 Turbo RARA) images were acquired using the following parameters: TE/TR, 43.67/7,600 ms; rare factor, 8; matrix size, 256 × 256; voxel size, 60 × 60 × 200 µm^3^; number of slices, 72; slice thickness, 200 µm; number of averages, 6; acquisition time, 25 min. Analysis was performed using IMARIS (Bitplane, v9.2). Mice were anesthetized under 3% isoflurane and positioned in an MRI-compatible head holder to minimize motion artifacts. Anesthesia was then maintained at 1.5% isoflurane in 100% O_2_ throughout imaging. The respiration rate was monitored using a pressure pad placed under the animal abdomen, and animal body temperature was maintained by a warming pad (37 °C) placed under the animal. The high-resolution in vivo T2-weighted images were oriented to Badhwar hippocampal atlas space^59^, corrected for noise^60^ and skull stripped using STAPLE algorithm^61^. The skull stripped brain volumes were corrected for B1 field inhomogeneity using N4 bias field correction algorithm and then registered to the Badhwar hippocampal atlas non-linearly using the Symmetric diffeomorphic image registration with cross-correlation (SyN) algorithm implemented in ANTs^62–64^. The third ventricle, fourth ventricle and lateral ventricle were combined as a single region of interest in atlas space and then transformed to individual in vivo T2-weighted image space using an inverse transform matrix and deformation map, which were generated during the forward registration. Using the registered region of interest (ventricle) as a prior label and the sample-specific T2-weighted image as a reference image, additional improvement in registration/segmentation was achieved using ANTs Atropos tool^65^. For each sample, final segmentation results were manually inspected for missing data registration. Using the brain mask, total intracranial volume was extracted with the FSL ‘fslstats’ tool, and using the segmented ventricle, total ventricle volume was also extracted with the fslstats tool.

To investigate group differences in ventricle volume, a general linear model was used. The independent between-group assessment was corrected for the effect of total intracranial volume. A post hoc test was conducted to further understand the sensitivity of ventricle size in terms of groupwise comparisons. The analysis was performed in SPSS (IBM, SPSS, version 27). To account for multiple comparisons across four groups, FDR correction using Benjamini–Hochberg criterion (*α* = 0.05) was used (pFDR < 0.05).

### Statistics and reproducibility

No statistical methods were used to predetermine sample sizes, but our sample sizes are similar to those reported in previous publications^23,39,66,67^. All statistical analysis and graph designs were performed using GraphPad Prism 9. Results in column graphs represent the mean ± s.e.m. Student’s *t*-test, one-way ANOVA and two-way ANOVA tests were performed as necessary. For all tests, an alpha value of 0.05 was used to determine statistical significance. Data distribution was assumed to be normal but this was not formally tested. Data collection and analysis were performed blind to the conditions of the experiments. No animals or data points were excluded, and outlier analysis was not performed.

### Reporting summary

Further information on research design is available in the Nature Research Reporting Summary linked to this article.

## Online content

Any methods, additional references, Nature Research reporting summaries, source data, extended data, supplementary information, acknowledgements, peer review information; details of author contributions and competing interests; and statements of data and code availability are available at 10.1038/s41593-022-01191-6.

## Supplementary information

Supplementary Information Supplementary Table 1

Reporting Summary

Supplementary Data 1 **Tau-seed interactome and tau monomer interactome**. Tau monomer interactome in PS19 mouse brains. Monomeric tau isolated from F17 from PS19 brain homogenates was subjected to quantitative mass spectrometry; *n* = 3. Tau-seed interactome in PS19 mouse brains. HMW tau seed isolated from F9 from PS19 brain homogenates was subjected to quantitative mass spectrometry; *n* = 3.

Supplementary Data 2 **DAVID functional annotation and interactome versus nominated intersection**. DAVID functional annotation clustering of tau-seed interactors. DAVID functional annotation chart of tau-seed interactors. DAVID functional annotation clustering of tau-monomer interactors. DAVID functional annotation chart of tau-monomer interactors. Synaptic interactors of the tau seed and monomeric fractions.

## Data Availability

Mass spectrometry data are deposited in the ProteomeXchange Consortium via the PRIDE^68–70^ partner repository under dataset identifiers PXD027451 and PXD027451. Source data are provided with this paper. All other numerical data are available from the corresponding author upon request.

## Source data

Source Data Fig. 1 Unprocessed western blots and/or gels.
Source Data Fig. 1 Statistical source data.
Source Data Fig. 2 Unprocessed western blots and/or gels.
Source Data Fig. 2 Statistical source data.
Source Data Fig. 3 Unprocessed western blots and/or gels.
Source Data Fig. 3 Statistical source data.
Source Data Fig. 4 Statistical source data.
Source Data Fig. 5 Unprocessed western blots and/or gels.
Source Data Fig. 5 Statistical source data.
Source Data Fig. 6 Statistical source data.
Source Data Fig. 7 Unprocessed western blots and/or gels.
Source Data Fig. 7 Statistical source data.
Source Data Extended Data Fig. 1 Statistical source data.
Source Data Extended Data Fig. 2 Statistical source data.
Source Data Extended Data Fig. 4 Unprocessed western blots and/or gels.
Source Data Extended Data Fig. 4 Statistical source data.
Source Data Extended Data Fig. 5 Unprocessed western blots and/or gels.
Source Data Extended Data Fig. 5 Statistical source data.
Source Data Extended Data Fig. 6 Unprocessed western blots and/or gels.
Source Data Extended Data Fig. 6 Statistical source data.
Source Data Extended Data Fig. 7 Statistical source data.
Source Data Extended Data Fig. 8 Statistical source data.
Source Data Extended Data Fig. 9 Statistical source data.
Source Data Extended Data Fig. 10 Statistical source data.
