# Supplementary material for: Bassoon contributes to tau-seed propagation and neurotoxicity
Source: Nat Neurosci. 2022 Nov 7;25(12):1597–607. doi: 10.1038/s41593-022-01191-6 (PMC9708566; doi:10.1038/s41593-022-01191-6)
Supplement: Supplementary file 1 — Supplementary Table 1 [file 41593_2022_1191_MOESM1_ESM.pdf]

nature neuroscience

Article

https://doi.org/10.1038/s41593-022-01191-6

Bassoon contributes to tau-seed propagation and neurotoxicity

In the format provided by the authors and unedited

Supplementary Information

Supplementary Table 1. Human AD and PSP cases used in this study.

Case	Disease	Cerad	Braak	Age	Sex	Race	PMD	Brain region	Assay
4	Control	-	-	63	F	W	22	MFG, PONS	SEC, Seeding. IF
39	Control	-	-	63	M	W	30-34	MFG, PONS	SEC, Seeding, IF
123	Control	-	-	80	F	W		MFG, PONS	SEC, Seeding
155	Control	-	-	72	M	W	24	MFG, PONS	SEC, Seeding
189	Control	-	-	71	F	W	17	MFG, PONS	SEC, Seeding
2338	AD	C	6	87	M	W	7	MFG	IF, Seeding
2344	AD	C	5	86	M	W	5	MFG	SEC, Seeding
2423	AD	B	6	89	F	W	7	MFG	IF, Seeding
2447	AD	C	6	65	M	W	11	MFG	IF, Seeding
2494	AD	C	4	81	F	W	15	MFG	SEC, Seeding
2495	AD	C	5	67	M	W	6	MFG	SEC, Seeding
1715	PSP	0		77	F	W	2	PONS	SEC, Seeding
1827	PSP	0		72	F	W	15	PONS	SEC, Seeding
2121	PSP	0	4	85	M	W	7	PONS	SEC, Seeding
2147	PSP	0	4	87	F	W	17	PONS	IF, Seeding
2159	PSP	0	0	74	F	W	20	PONS	IF, Seeding
2421	PSP	0	4	66	M	W	9.5	PONS	IF, Seeding
