# Supplementary material for: Bassoon contributes to tau-seed propagation and neurotoxicity
Source: Nat Neurosci. 2022 Nov 7;25(12):1597–607. doi: 10.1038/s41593-022-01191-6 (PMC9708566; doi:10.1038/s41593-022-01191-6)
Supplement: Supplementary file 2 — Reporting Summary [file 41593_2022_1191_MOESM2_ESM.pdf]

nature portfolio

Corresponding author(s): Cristian Lasagna-Reeves

Last updated by author(s): 2022/09/22

Reporting Summary

Nature Portfolio wishes to improve the reproducibility of the work that we publish. This form provides structure for consistency and transparency in reporting. For further information on Nature Portfolio policies, see our Editorial Policies and the Editorial Policy Checklist.

Statistics

For all statistical analyses, confirm that the following items are present in the figure legend, table legend, main text, or Methods section.

n/a	Confirmed	
☐	☒	The exact sample size (*n*) for each experimental group/condition, given as a discrete number and unit of measurement
☐	☒	A statement on whether measurements were taken from distinct samples or whether the same sample was measured repeatedly
☐	☒	The statistical test(s) used AND whether they are one- or two-sided<br>*Only common tests should be described solely by name; describe more complex techniques in the Methods section.*
☒	☐	A description of all covariates tested
☐	☒	A description of any assumptions or corrections, such as tests of normality and adjustment for multiple comparisons
☐	☒	A full description of the statistical parameters including central tendency (e.g. means) or other basic estimates (e.g. regression coefficient) AND variation (e.g. standard deviation) or associated estimates of uncertainty (e.g. confidence intervals)
☐	☒	For null hypothesis testing, the test statistic (e.g. *F*, *t*, *r*) with confidence intervals, effect sizes, degrees of freedom and *P* value noted<br>*Give P values as exact values whenever suitable.*
☒	☐	For Bayesian analysis, information on the choice of priors and Markov chain Monte Carlo settings
☒	☐	For hierarchical and complex designs, identification of the appropriate level for tests and full reporting of outcomes
☐	☒	Estimates of effect sizes (e.g. Cohen's *d*, Pearson's *r*), indicating how they were calculated

Our web collection on statistics for biologists contains articles on many of the points above.

Software and code

Policy information about availability of computer code

Data collection	Epifluorescent microscopy data was collected using Leica Application Suite X 3.6.0.20104 on a Leica DMi8 microscope<br>Confocal Imaging was collected using NIS Elements 5.21.03 on a Nikon A1R laser scanning confocal microscope<br>Flow cytometry data was acquired using BD FACS Diva (v8.0)<br>For electrophysiology measurements, Clampex and Clampfit (v11.1) were used.
Data analysis	Imaging data were processed and analyzed using Image J (NIH, v1.53i) and plugins (e.g. Flynotyper plug-in), IMARIS (Bitplane, v9.2), and Zerenestacker (v1.01). For cytometry analysis FlowJo (v10.0) was used. Data was statistically analyzed using GraphPad Prism (v9.0).

For manuscripts utilizing custom algorithms or software that are central to the research but not yet described in published literature, software must be made available to editors and reviewers. We strongly encourage code deposition in a community repository (e.g. GitHub). See the Nature Portfolio guidelines for submitting code & software for further information.

Data

Policy information about availability of data

All manuscripts must include a data availability statement. This statement should provide the following information, where applicable:

- Accession codes, unique identifiers, or web links for publicly available datasets
- A description of any restrictions on data availability
- For clinical datasets or third party data, please ensure that the statement adheres to our policy

Mass spectrometry data have been deposited to the ProteomeXchange Consortium via the PRIDE partner repository with the dataset identifier PXD027451 and 10.6019/PXD027451. Raw data can be found in Source Data. Uncropped western blots can be found in Source Data Figures. All other numerical data are available from the corresponding authors upon request.

Field-specific reporting

Please select the one below that is the best fit for your research. If you are not sure, read the appropriate sections before making your selection.

☒ Life sciences ☐ Behavioural & social sciences ☐ Ecological, evolutionary & environmental sciences

For a reference copy of the document with all sections, see nature.com/documents/nr-reporting-summary-flat.pdf

Life sciences study design

All studies must disclose on these points even when the disclosure is negative.

Sample size	No statistical methods were performed to predetermine sample size. Sample sizes were chosen based on literature to determine biochemical, behavioral, electrophysiological and MRI changes (Lasagna-Reeves C.A.,et al. Neuron; Patel H.,et al. NBA; Jadhav V.,et al. Molecular Neurodegeneration; Yoshiyama Y.,et al. Neuron). The sample size (n) of each experiment is provided in the figure legends.
Data exclusions	No data were excluded from any analysis performed.
Replication	All experiments were repeated at least three times, and the results are reproducible. Imaging, electrophysiology and behavioral experiments were performed using different cohorts of animals. The results are consistent and robust.
Randomization	Animals were randomly assigned to the treatment and experimental groups with matched age and sex in the following experiments: biochemistry, electrophysiology, behavior and MRI. Animal brain sections used for imaging were also selected randomly.
Blinding	All the experiment and analysis were performed blindly.

Reporting for specific materials, systems and methods

We require information from authors about some types of materials, experimental systems and methods used in many studies. Here, indicate whether each material, system or method listed is relevant to your study. If you are not sure if a list item applies to your research, read the appropriate section before selecting a response.

Materials & experimental systems

n/a	Involved in the study
☐	☒ Antibodies
☐	☒ Eukaryotic cell lines
☒	☐ Palaeontology and archaeology
☐	☒ Animals and other organisms
☒	☐ Human research participants
☒	☐ Clinical data
☒	☐ Dual use research of concern

Methods

n/a	Involved in the study
☒	☐ ChIP-seq
☐	☒ Flow cytometry
☐	☒ MRI-based neuroimaging

Antibodies

Antibodies used	Primary antibodies: Biotinylated mouse IgG1 (BioLegend, 400104, 2µg of antibody every 100ng of total tau), biotinylated HT7 (Thermofisher, MN1000b, 2µg of antibody every 100ng of total tau), HT7 (Thermofisher, MN1000, 1:1000), anti-Bassoon (Millipore, ABN255, 1:1000), anti-GFP (Abcam, ab1218, 1:1000), MC-1 (Peter Davies, 1:1000) , anti-total tau (DAKO, A0024, 1:1000) , PHF1 (Peter Davies, 1:1000), p-tau Thr231 (Millipore, MAB5450, 1:1000), anti-Actin (Abcam, ab8227, 1:2000), Vinculin (Sigma, V9131, 1:1000), anti-GFAP (Sigma-Aldrich, G3893, 1:100) and anti-IBA1 (Wako, 019-19741, 1:100), 6X-His (Thermo Fisher MA1-135, 1:300), PSD95 (Abcam, ab2723, 1:100), Synapsin-1 (Abcam, ab64581, 1:100).<br>Secondary antibodies: goat anti-rabbit Alexa Fluor 488 (Invitrogen, A11008, 1:200), goat anti-mouse Alexa Fluor 488 (Invitrogen, A32723, 1:200), goat anti-rabbit Alexa Fluor 568 (Invitrogen, A11036, 1:200), goat anti-mouse Alexa Fluor 568 Invitrogen, A11031, 1:200), goat anti-mouse HRP conjugated (Invitrogen, 626820, 1:5000), goat anti-rabbit HRP conjugated (Invitrogen, 31460, 1:5000).
Validation	1. Validation for immunoprecipitation by the company and/or studies cited on company's websites:<br>biotinylated HT7 (Thermofisher, MN1000b) https://www.thermofisher.com/antibody/product/Tau-Antibody-clone-HT7-Monoclonal/MN1000B<br>Biotinylated mouse IgG1 (BioLegend, 400104) https://www.biolegend.com/fr-ch/products/biotin-mouse-igg1-kappa-isotype-ctrl-1405<br>2. Validation for Western blots by the company and/or studies cited on company's websites:<br>HT7 (Thermofisher, MN1000) https://www.thermofisher.com/antibody/product/Tau-Antibody-clone-HT7-Monoclonal/MN1000<br>anti-Bassoon (Millipore, ABN255) https://www.emdmillipore.com/US/en/product/Anti-Bassoon-Antibody,MM_NF-ABN255<br>anti-total tau (DAKO, A0024) https://www.labome.com/product/Dako/A0024.html<br>p-tau Thr231 (Millipore, MAB5450) https://www.emdmillipore.com/US/en/product/Anti-Tau-Antibody-phosphoThreonine-231-clone-PHF-6,MM_NF-MAB5450?ReferrerURL=https%3A%2F%2Fwww.google.com%2F

anti-Actin (Abcam, ab8227) https://www.abcam.com/beta-actin-antibody-ab8227.html
Vinculin (Sigma, V9131) https://www.sigmaaldrich.com/US/en/product/sigma/v9131
3. Validation for immunostaining by the company and/or studies cited on company's websites:
anti-Bassoon (Millipore, ABN255) https://www.emdmillipore.com/US/en/product/Anti-Bassoon-Antibody,MM_NF-ABN255anti-GFP (Abcam, ab1218) https://www.abcam.com/gfp-antibody-9f9f9-ab1218.html
anti-GFAP (Sigma-Aldrich, G3893) https://www.emdmillipore.com/US/en/product/Anti-Glial-Fibrillary-Acidic-Protein-Antibody-clone-GA5,MM_NF-MAB360
anti-IBA1 (Wako, 019-19741) https://labchem-wako.fujifilm.com/us/product/detail/W01W0101-1974.html
6X-His (Thermo Fisher MA1-135) https://www.thermofisher.com/antibody/product/6x-His-Tag-Antibody-clone-4E3D10H2-E3-Monoclonal/MA1-135
PSD95 (Abcam, ab2723) https://www.abcam.com/psd95-antibody-6g6-1c9-synaptic-marker-ab2723.html
Synapsin-1 (Abcam, ab64581) https://www.abcam.com/synapsin-i-antibody-synaptic-marker-ab64581.html
anti-total tau (DAKO, A0024) https://www.labome.com/product/Dako/A0024.html

4. MC1 and PHF1 (Peter Davis) have been validated and published previously (Petry F, et al. PLOS One)

Eukaryotic cell lines

Policy information about cell lines

Cell line source(s)	HEK293T (ATCC CRL-3216), TauRD P301S FRET biosensor (ATCC CRL-3275)
Authentication	None of these cell lines have been authenticated.
Mycoplasma contamination	HEK293T for transfection: not tested for mycoplasma contamination.<br>HEK293 biosensor: not tested for mycoplasma contamination.<br>HEK293T for AAV production: tested negative for mycoplasma contamination (analisis from Vectorbuilder).
Commonly misidentified lines (See ICLAC register)	No misindentified cell lines were used in the study.

Animals and other organisms

Policy information about studies involving animals; ARRIVE guidelines recommended for reporting animal research

Laboratory animals	For biochemistry experiments: PS19 and WT mice were sacrificed at 4 and 9 months.<br>For electrophysiology: 6 months old PS19 and WT littermates were euthanized.<br>For behavioral test and MRI: 9 months old PS19 and WT mice were used.<br>For propagation experiments: 12 weeks old C57BL/6J WT strains were used for AAV injections and sacrificed at 24 weeks.<br>For all the experiments, both male and female mice from each strain were used.
Wild animals	No wild animals were used in this study.
Field-collected samples	No field-collected animals were used in this study.
Ethics oversight	The study received ethical approval from IUSM Institutional Animal Care and Use Committee. All mice procedures were performed in accordance with the Guide for the Care and Use of Laboratory Animals (National Institutes of Health, Bethesda, MD). Mice were also anesthetized and euthanized according to IUSM Institutional Animal Care and Use Committee-approved procedures.

Note that full information on the approval of the study protocol must also be provided in the manuscript.

Flow Cytometry

Plots

Confirm that:

☒ The axis labels state the marker and fluorochrome used (e.g. CD4-FITC).

☒ The axis scales are clearly visible. Include numbers along axes only for bottom left plot of group (a 'group' is an analysis of identical markers).

☒ All plots are contour plots with outliers or pseudocolor plots.

☒ A numerical value for number of cells or percentage (with statistics) is provided.

Methodology

Sample preparation	Tau RD P301S FRET Biosensor (ATCC CRL-3275™) cells were transfected with seeding material using Lipofectamine 2000, incubated for 2 days, and run without fixation.
Instrument	BD LSRFortessa X-20
Software	Collection: BD FACS Diva (v8.0)<br>Analysis: FlowJo (v10.0)

Cell population abundance	Typical cell populations at each gating stage are as follows:<br>Cells: 70%-90%, Single cells: 70%-90%, FRET: 0-50%
Gating strategy	Cells were gated on a FSC-A vs SSC-A plot. Then single cells were gated on a SSC-W vs SSC-H plot. FRET positive cells were gated on a BV421-A vs BV510-A plot after compensation. The FRET-positive population lies in the region with same BV421-A intensity, but higher BV510-A intensity, with the boundary between positive and negative determined by excluding the signal from a negative control.

☒ Tick this box to confirm that a figure exemplifying the gating strategy is provided in the Supplementary Information.

Magnetic resonance imaging

Experimental design

Design type	not fMRI
Design specifications	The imaging study includes anatomical scans using T2-weighted imaging.
Behavioral performance measures	This is a structural imaging study. The animals were under anesthetization and no behavioral tasks or neuronal stimulations were delivered to the animals.

Acquisition

Imaging type(s)	T2-weighted anatomical imaging.
Field strength	9.4T
Sequence & imaging parameters	T2 Turbo RARA, TE/TR = 43.67/7600 ms, Rare factor = 8, matrix size = 256x256, voxel size =60x60x200 µm3, number of slices =72, slice thickness = 200 µm, number of averages = 6 and acquisition time =25 minutes.
Area of acquisition	whole brain

Diffusion MRI ☐ Used ☒ Not used

Preprocessing

Preprocessing software	The high resolution in-vivoT2-W images were oriented to Badhwar hippocampal atlas space, corrected for noise and skull stripped using STAPLE algorithm. The skull stripped brain volumes were corrected for B1 field inhomogeneity using N4 bias field correction algorithm and then non-linearly registered to Badhwar hippocampal atlas using Symmetric diffeomorphic image registration with cross-correlation (SyN) algorithm implemented in ANTs registration tool.
Normalization	non-linearly registered to Badhwar hippocampal atlas using Symmetric diffeomorphic image registration with cross-correlation (SyN) algorithm implemented in ANTs registration tool
Normalization template	Badhwar hippocampal atlas
Noise and artifact removal	corrected for noise and skull stripped using STAPLE algorithm
Volume censoring	only one volume, no volume censoring

Statistical modeling & inference

Model type and settings	N/A, not fMRI
Effect(s) tested	N/A, not fMRI

Specify type of analysis: ☐ Whole brain ☒ ROI-based ☐ Both

Anatomical location(s)	Third ventricle, fourth ventricle and lateral ventricle were combined as a single region of interest (ROI) in atlas space and then transformed to individual in-vivo T2-W image space using inverse transform matrix and deformation map, which were generated during the forward registration.
Statistic type for inference<br>(See Eklund et al. 2016)	Using registered ROI (ventricle) as prior label and sample specific T2-W image as a reference image, additional improvement in registration/segmentation was achieved using ANTs Atropos tool [7]. For each sample, final segmentation results were manually inspected for miss registration. Using brain mask, total intra cranial volume (TICV) was extracted using FSL "fslstats" tool and using segmented ventricle, total ventricle volume was also extracted using FSL "fslstats" tool.
Correction	To investigate group differences in ventricle volume, general linear model (GLM) was used. The independent between-group (WT, WTT, PS19 and PS19T) assessment was corrected for the effect of TICV. A post-hoc test was conducted to further understand the sensitivity of ventricle size in terms of group-wise comparisons. The analysis was performed in SPSS (IBM,

SPSS, Version 27). To account for multiple comparisons across 4 groups, false discovery rate (FDR) correction using Benjamini-Hochberg criterion (α=0.05) was used (P_{FDR} < 0.05).

Models & analysis

n/a	Involved in the study
☒	☐ Functional and/or effective connectivity
☒	☐ Graph analysis
☒	☐ Multivariate modeling or predictive analysis
