# Supplementary figures and images for: Bassoon contributes to tau-seed propagation and neurotoxicity
Source: Nat Neurosci. 2022 Nov 7;25(12):1597–607. doi: 10.1038/s41593-022-01191-6 (PMC9708566; doi:10.1038/s41593-022-01191-6)

**h**

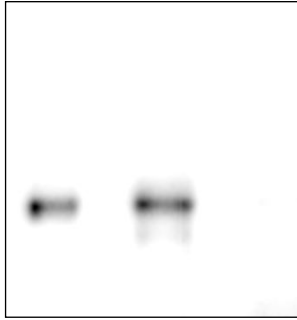

Uncropped and unprocessed western blot (WB) scans for Figure 1h.

Supplement: Source Data Fig. 1 — Unprocessed western blots and/or gels. [file 41593_2022_1191_MOESM5_ESM.pdf]

**h**

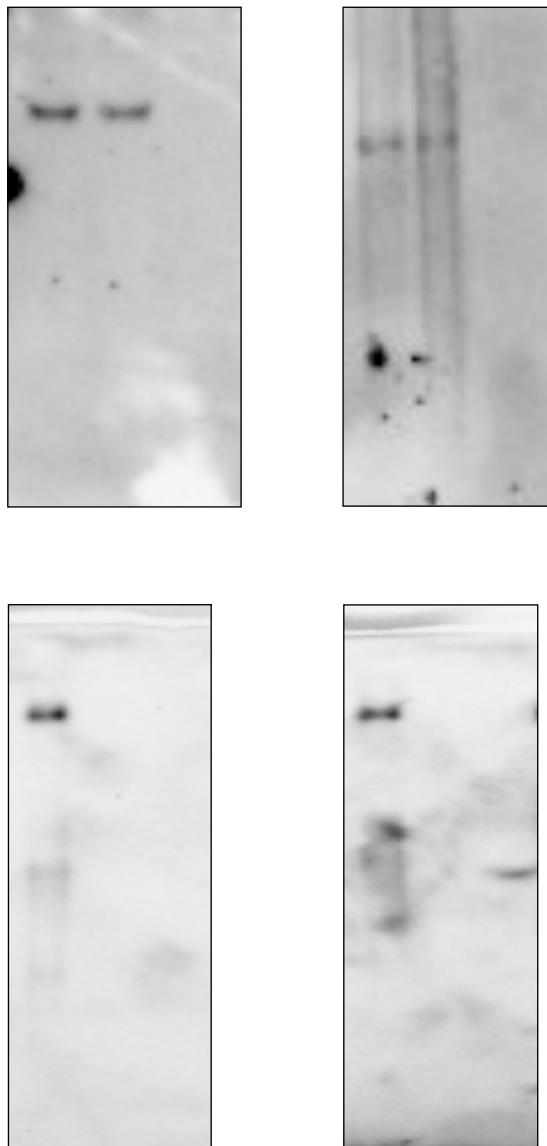

Uncropped and unprocessed western blot (WB) scans for Figure 2h.

Supplement: Source Data Fig. 2 — Unprocessed western blots and/or gels. [file 41593_2022_1191_MOESM7_ESM.pdf]

**b**

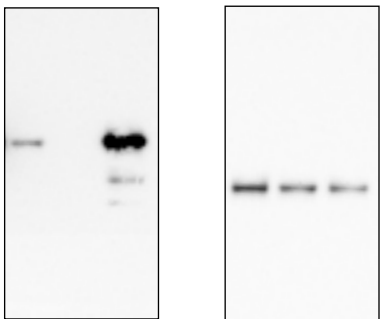

**d**

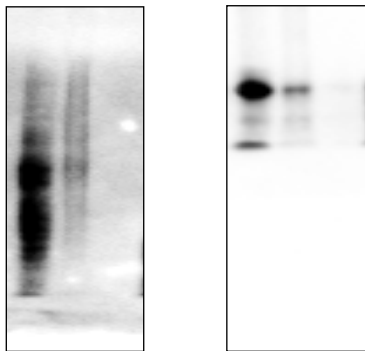

Uncropped and unprocessed western blot (WB) scans for Figure 3b and 3d.

Supplement: Source Data Fig. 3 — Unprocessed western blots and/or gels. [file 41593_2022_1191_MOESM9_ESM.pdf]

g

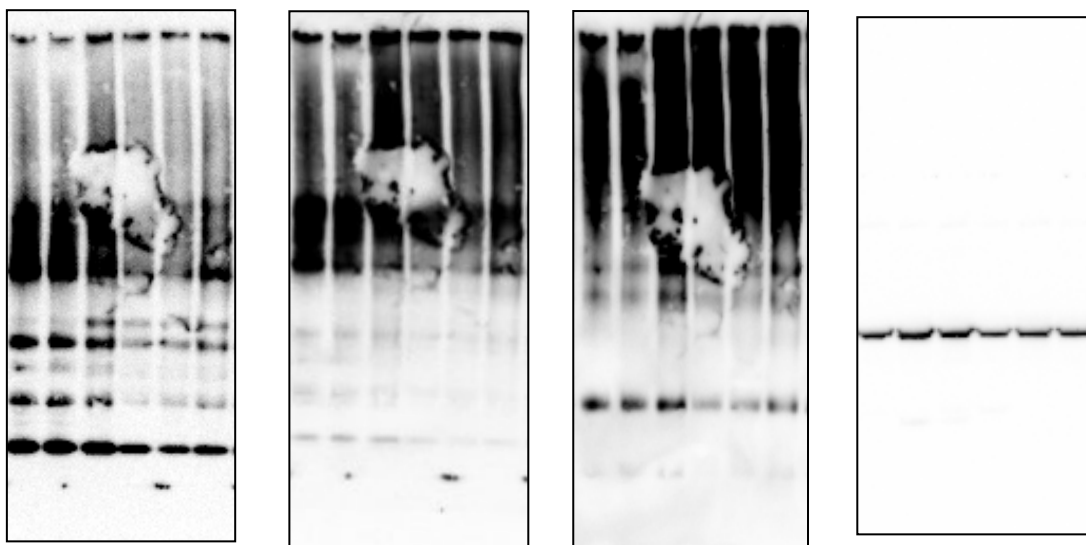

Uncropped and unprocessed western blot (WB) scans for Figure 7g.

Supplement: Source Data Fig. 7 — Unprocessed western blots and/or gels. [file 41593_2022_1191_MOESM15_ESM.pdf]

**a**

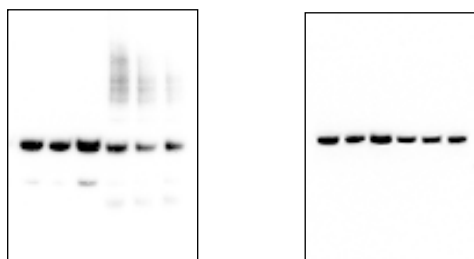

**h**

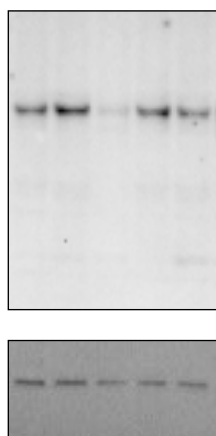

**i**

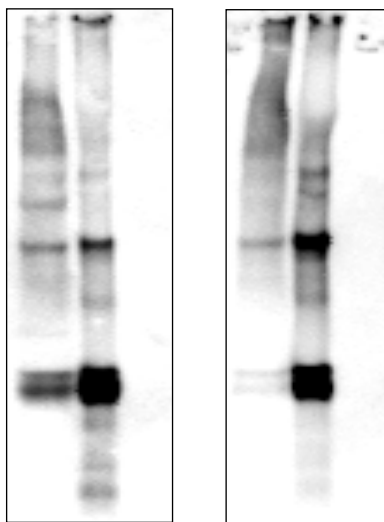

**j**

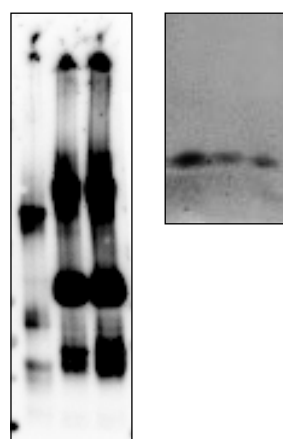

**k**

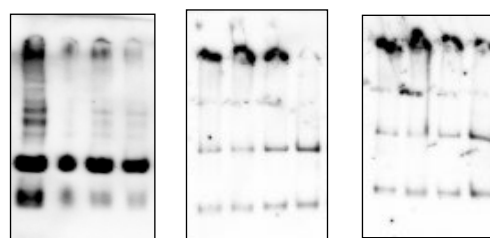

Uncropped and unprocessed western blot (WB) scans for Extended Data Figure 4a, 4h, 4i, 4j, and 4k.

Supplement: Source Data Extended Data Fig. 4 — Unprocessed western blots and/or gels. [file 41593_2022_1191_MOESM19_ESM.pdf]

**a**

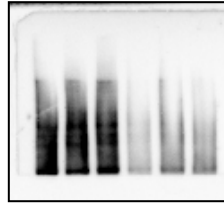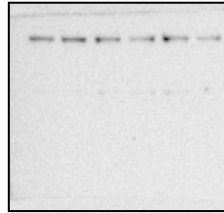

Uncropped and unprocessed western blot (WB) scans for Extended Data Figure 5a.

Supplement: Source Data Extended Data Fig. 5 — Unprocessed western blots and/or gels. [file 41593_2022_1191_MOESM21_ESM.pdf]

**a**

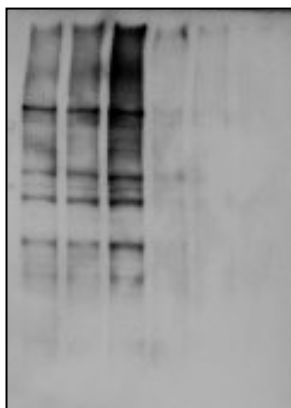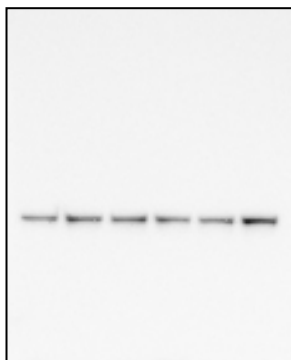

Uncropped and unprocessed western blot (WB) scans for Extended Data Figure 6a.

Supplement: Source Data Extended Data Fig. 6 — Unprocessed western blots and/or gels. [file 41593_2022_1191_MOESM23_ESM.pdf]
