# Supplementary material for: Bassoon contributes to tau-seed propagation and neurotoxicity
Source: Nat Neurosci. 2022 Nov 7;25(12):1597–607. doi: 10.1038/s41593-022-01191-6 (PMC9708566; doi:10.1038/s41593-022-01191-6)
Supplement: Source Data Fig. 5 — Unprocessed western blots and/or gels. [file 41593_2022_1191_MOESM12_ESM.pdf]

f

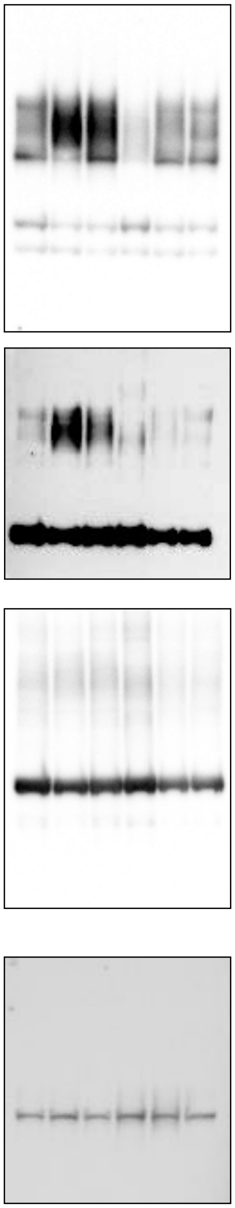

m

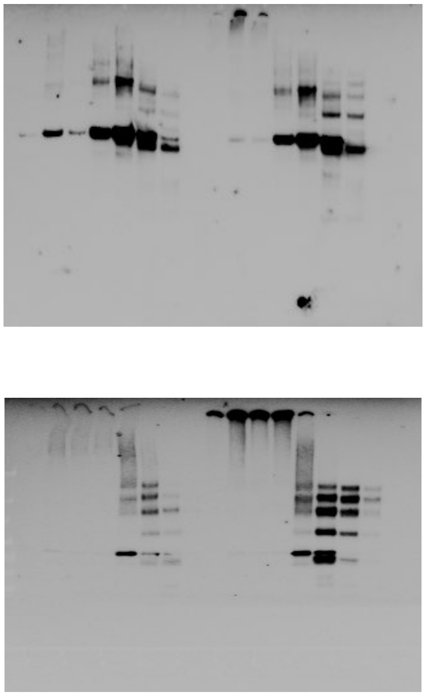

Uncropped and unprocessed western blot (WB) scans for Figure 5f and 5m. Figure (m) was cropped and reoriented for easier interpretation.
